# Synergetic Catalytic Effect between Ni and Co in Bimetallic Phosphide Boosting Hydrogen Evolution Reaction

**DOI:** 10.3390/nano14100853

**Published:** 2024-05-14

**Authors:** Xiaohan Wang, Han Tian, Libo Zhu, Shujing Li, Xiangzhi Cui

**Affiliations:** 1 Shanghai Institute of Ceramics, Chinese Academy of Sciences, Shanghai 200050, China; 18063069758@163.com (X.W.); tianhan@mail.sic.ac.cn (H.T.); 15562606956@163.com (L.Z.); 2 School of Chemistry and Materials Science, Hangzhou Institute for Advanced Study, University of Chinese Academy of Sciences, Hangzhou 310024, China; lishujing@mail.sic.ac.cn; 3 Center of Materials Science and Optoelectronics Engineering, University of Chinese Academy of Sciences, Beijing 100049, China

**Keywords:** hydrogen evolution reaction, bimetallic phosphide, synergetic effect, water splitting

## Abstract

The application of electrochemical hydrogen evolution reaction (HER) for renewable energy conversion contributes to the ultimate goal of a zero-carbon emission society. Metal phosphides have been considered as promising HER catalysts in the alkaline environment, which, unfortunately, is still limited owing to the weak adsorption of H* and easy dissolution during operation. Herein, a bimetallic NiCoP-2/NF phosphide is constructed on nickel foam (NF), requiring rather low overpotentials of 150 mV and 169 mV to meet the current densities of 500 and 1000 mA cm^−2^, respectively, and able to operate stably for 100 h without detectable activity decay. The excellent HER performance is obtained thanks to the synergetic catalytic effect between Ni and Co, among which Ni is introduced to enhance the intrinsic activity and Co increases the electrochemically active area. Meanwhile, the protection of the externally generated amorphous phosphorus oxide layer improves the stability of NiCoP/NF. An electrolyser using NiCoP-2/NF as both cathode and anode catalysts in an alkaline solution can produce hydrogen with low electric consumption (overpotential of 270 mV at 500 mA cm^−2^).

## 1. Introduction

Developing renewable energy sources have drawn significant interest from the scientific and industrial communities due to increasing consumption of fossil fuels and environmental pollution [1,2,3]. Hydrogen energy is one of the most valuable renewable energy sources thanks to its high energy density and cleaning. One way to effectively use hydrogen energy is through the electrocatalytic hydrogen evolution reaction (HER) [4]. Unlike the relatively easy adsorption of active hydrogen in an acidic medium, the alkaline HER reaction kinetics are sluggish, requiring higher potentials and the application of expensive catalysts to lower the reaction energy barrier [5]. So far, the benchmark catalysts are still Pt or Pt-based catalysts [1,6]; however, small reserves and high costs are the serious impediments to their large-scale development. Consequently, there is a need to develop catalysts based on non-precious metals that have high catalytic activity and good durability [7,8,9].

Since metal phosphides (FeP [10], CoP [4], NiP [11], NiCoP [12,13]……) exhibit excellent electrical conductivity and fast charge transfer capability, they have recently shown great potential for hydrogen production in an alkaline environment [14,15,16]. Importantly, the moderate binding energy between P and the hydrogen species can inhibit the excessive adsorption on pure metals and enhance the desorption of H_2_ [17]. However, the development of phosphides is still limited for the following two reasons. On the one hand, the weak adsorption of H* on the catalyst surface limits the improvement to the HER performance of non-precious metal phosphides. On the other hand, the surface reconstruction of metal phosphide is a frequently observed phenomenon in the alkaline HER process, and the subsequent dissolution results in poor stability. Therefore, huge challenges still remain to obtain metal phosphides with satisfactory HER activity and durability.

Recent studies have shown that the addition of heterogeneous metals can alter the electronic structure of monometallic phosphides, thereby improving their catalytic efficiency [18,19,20]. On this basis, we constructed a bimetallic NiCoP heterojunction to modulate the intrinsic electronic distribution and enhance the H* coverage on the catalyst surface during HER under alkaline conditions, making NiCoP a promising candidate for efficient HER catalysis.

With the use of phosphating and hydrothermal processes, the novel NiCoP-2/NF bimetallic phosphide was successfully prepared. Based on SEM images, electrochemical results, and analog circuit measurements, it was shown that the addition of Co element during the preparation process gradually transforms the surface nanosheets into dense nanowires, and this morphology change is beneficial for improving the specific surface area of the catalyst. Meanwhile, the addition of Ni element can enhance the H* adsorption capacity on the catalyst surface, which further optimizes the intrinsic activity of the catalyst. Combined with the above experimental results and analysis, NiCoP-2/NF with the Co-Ni adding ratio of 1:1 has the optimized HER activity, which can act as an effective HER electrocatalyst at industrial-scale current densities (500 mA cm^−2^ and 1000 mA cm^−2^), with required overpotentials of only 150 and 169 mV. At the same time, the externally generated amorphous phosphorus oxide layer protects the catalyst and endows the as-prepared NiCoP-2/NF with good stability. Combining the aforementioned two aspects, the integrated two-electrode system with NiCoP-2/NF as the anode and cathode catalysts achieves 500 mA cm^−2^ at 2.13 V, which is about 270 mV lower than that of 20% Pt/C-NF||RuO_2_-NF, and maintains good performance even after 100 h of continuous operation.

## 2. Materials and Methods

### 2.1. Materials

Sinopharm Group (Shanghai, China) provided the following products: Ni(NO_3_)_2_·6H_2_O, urea, and hydroxychloric acid (HCl). Aladdin (Shanghai, China) provided the following products: RuO_2_ (99% metal basis), Co(NO_3_)_2_·6H_2_O, Ammonium fluoride (NH_4_F), and Sodium hypophosphite (NaH_2_PO_2_). Shanghai Titan Scientific Co., Ltd. (Shanghai, China) provided the sodium hydroxide (KOH). Dupont China Holding Co., Ltd. (Shanghai, China) was the supplier of the Nafion D-520. Shanghai HEPHAS Energy Equipment Co., Ltd. (Shanghai, China) provided the XC-72 and commercial 20 wt.% Pt/C. Cyber Electric Co., Ltd. (Hong Kong, China) supplied the Nickel foam (1.0 mm in thickness, 0.1 mm in aperture, and 97.2% porosity). Nothing was purified further.

### 2.2. Material Synthesis

#### 2.2.1. Preparation of NiCoP/NF

In order to get rid of any potential oils on the surface and oxidized layers, the nickel foam (NF) was cleaned in stages using ethanol, 1 M hydrochloric acid, and deionized water. It was then ultrasonicated for 10 min. Next, the prepared nickel foams (2 cm × 3.5 cm) were poured into and fully submerged in as-prepared solution that contained deionized water (60 mL), Ni(NO_3_)_2_·6H_2_O (3 mmol), Co(NO_3_)_2_·6H_2_O (3 mmol), NH_4_F (4 mmol), and urea (10 mmol). The hydrothermal reactor was then maintained at 120 °C for 6 h. The substrate was removed, cleaned, cooled naturally, and dried. In a lengthy crucible, the precursor was on the downstream side and the NaH_2_PO_2_ was on the upstream side. After that, it was heated for four hours at a rate of 2 °C per minute at 325 °C in an Ar atmosphere. After cooling in Ar, NiCoP-2/NF was produced. In addition, samples were prepared with Co source and Ni source additions of 4 mmol + 2 mmol and 2 mmol + 4 mmol, named NiCoP-1/NF and NiCoP-3/NF, respectively.

#### 2.2.2. Synthesis of Other NF-Based Catalysts with Ni, CoP, 20% Pt/C, or RuO_2_

Similar to NiCoP/NF, the precursors of NiP/NF and CoP/NF were generated without the addition of Co(NO_3_)_2_·6H_2_O and Ni(NO_3_)_2_·6H_2_O, respectively.

To make the ink solution, Pt/C (10 mg of 20 wt%) was dissolved in 970 μL of isopropanol using ultrasonics: after that, Nafion solution (30 μL of 10%) was added. The 20% Pt/C-NF is then obtained by uniformly dropping 100 μL ink to 1 cm^2^ of nickel foam (1 mg cm^−2^).

RuO_2_-NF was prepared in the same way as 20% Pt/C-NF, except that the synthesis was changed from Pt/C to the addition of RuO_2_ (1 mg cm^−2^).

### 2.3. Electrochemical Measurements

Electrochemical tests were carried out at room temperature using VSP-300 (BioLogic, Seyssinet-Pariset, France) under a three-electrode system. CoP/NF, NiP/NF, NiCoP-1/NF, NiCoP-2/NF, NiCoP-3/NF, 20% Pt/C-NF, and RuO_2_-NF with geometrical areas of 0.25 cm^2^ were prepared as working electrodes in an electrolytic cell containing 1 M KOH solution, while carbon rods were used as counter electrodes. Ag/AgCl or Hg/HgO were used as a reference electrode for HER or OER, respectively. The loading amounts of as-prepared catalysts are 15.2 mg cm^−2^ for CoP/NF, 5.14 mg cm^−2^ for NiCoP-1/NF, 6.27 mg cm^−2^ for NiCoP-2/NF, 4.76 mg cm^−2^ for NiCoP-3/NF, and 5.63 mg cm^−2^ for NiP/NF. The electrode voltages included in the test results are for reversible hydrogen electrodes, and the conversion equation is: E (vs. RHE) = E (vs. Ag/AgCl) + 0.196 + 0.0591 × pH; E (vs. RHE) = E (vs. Hg/HgO) + 0.095 + 0.0591 × pH. LSV tests were performed with a sweep rate of 1 mV s^−1^, and iR compensation was used in some of the results, which are labeled in the main text. The Tafel data were obtained by converting LSV data. The Operando EIS was tested with an AC amplitude of 5 mV and a frequency range of 10^−2^ to 10^5^ Hz. The double-layer capacitance (C_dl_) values were obtained by varying the scanning speed in the CV curves in the non-Faraday region (30–130 mV) and were calculated to give the electrochemical surface area (ECSA) of the different catalysts. By varying the scan rate of the CV curves in the non-Faraday region (30–130 mV), the value of the double-layer capacitance (C_dl_) can be obtained by the formula C_dl_ = Δj/(2v), and the electrochemical surface area (ECSA) of the different catalysts can be calculated by the formula ECSA = C_dl_/C_f_ (C_f_ = 0.04 mF/cm^2^) The masses of NF are weighed before and after the preparation of NiCoP catalyst. Then, the difference of the above masses is divided by the NF area (2 cm × 3.5 cm) to obtain the corresponding mass loading. Next, the current values (A cm^−2^) derived from the LSV tests are divided by the mass loading for as-prepared catalysts to obtain the mass activity (A g^−1^). In addition, by controlling the dropping amounts of ink, the mass loadings of 20% Pt/C-NF and RuO_2_-NF are 1 mg cm^−2^ and 1 mg cm^−2^, respectively. Stirring is used in all the tests of LSV curves and i-t curves in electrochemical processes.

### 2.4. Exchange Current Density

Tafel curves were derived by combining the overpotential and current density data of the catalyst in the electrochemical reaction. The obtained Tafel curves were linearly fitted and the intersection of the linearly fitted data with the x-axis was derived, the value of which is lgJ_0_.

$$\eta =a+blgJ_{0}$$

η (V) is the overpotential and J_0_ is the exchange current density. b and a are the slopes and y-axis intercepts obtained from a linear fit of the Tafel curve.

### 2.5. Active Site Number

CV tests were performed in neutral solutions using a three-electrode system. During the reaction, all the catalysts prepared did not show any significant redox peaks, which indicates that the number of active sites on the surface of the catalysts is positively proportional to the integrated voltammetric charges. The number of active sites can be expressed by the following equation:

$$n=\frac{Q}{2F}$$

Q is the total charge in the CV curve and F is the Faraday efficiency.

### 2.6. TOF Calculation

Assuming that all active sites on the catalyst surface are exposed to solution.

$$TOF=\frac{1}{2}\frac{I}{nF}$$

I (A) denotes the current during the reaction, n (mol) denotes the density of active sites, F is the Faraday constant, and the coefficient 1/2 denotes that two electrons are required for the production of one hydrogen molecule.

## 3. Results and Discussion

### 3.1. Synthesis and Characterizations

**Figure 1:**
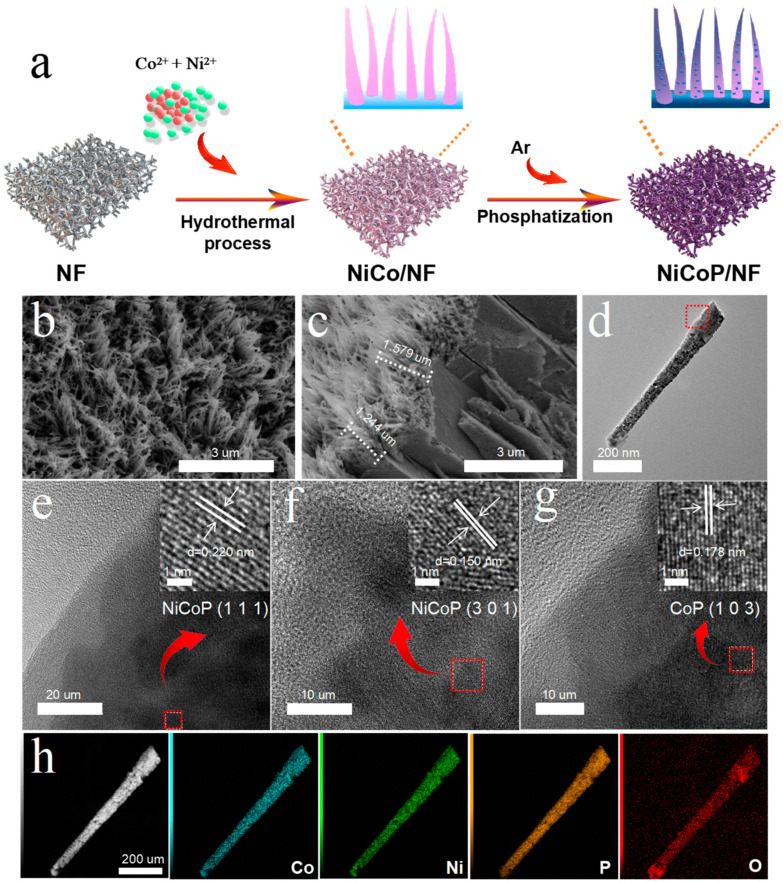
(**a**) Fabrication schematic illustration of NiCoP/NF. SEM images (**b**,**c**), TEM images (**d**), HRTEM images (**e**–**g**), and EDS elemental mappings (**h**) of NiCoP-2/NF.

In this research, thermal phosphating was used after hydrothermal treatment to create the NiCoP/NF. Utilizing Ni(NO_3_)_2_ and Co(NO_3_)_2_ as Ni and Co sources, respectively, the nanowires were produced in-situ on NF with high porosity and conductivity (Figure 1a). Then, NiCoP/NF can be synthesized by the phosphating of precursors using NaH_2_PO_2_ under low temperatures. The different Ni/Co ratios were used to make NiCoP-1/NF, NiCoP-2/NF, and NiCoP-3/NF, respectively. The different catalyst loadings have been included in Table S1.

Figure 1b,c depict the nanowire array shape of NiCoP-2/NF obtained from scanning electron microscopy (SEM) images. The average length of the array for NiCoP-2/NF is 1.4 um. In comparison, NiCoP-1/NF also appears to have a similar structure except for the longer nanowires (Figure S1), which proves that the addition of Ni source can decrease the length of the nanowires. With the further increase in the Ni adding amount, the nanowires disappear and the surface of NiCoP-3/NF is fully laid with many dense and uniform nanosheets (Figure S2). Furthermore, SEM images of the control samples are also shown in Figures S3 and S4, with the morphologies of the nanoplates (NiP/NF) and nanowires (CoP/NF), respectively. The morphology information was further examined using transmission electron microscopy (TEM) studies, which confirmed that NiCoP-2/NF is made up of many nanowires (Figure 1d). The high-resolution TEM (HRTEM) of a single nanowire shows lattice spacings of 0.220 nm, 0.150 nm, and 0.179 nm, which well correspond to the (111) plane, (301) plane of NiCoP, and (103) plane of CoP, respectively (Figure 1e–g). On the nanowires, Co, Ni, and P are evenly dispersed, while the broader O signal is due to the externally generated amorphous phosphorus oxide layer (Figure 1h). In addition, the Inductively Coupled Plasma (ICP) and energy dispersion spectrum (EDS), as shown in Figure S5, prove that the Ni/Co ratio of NiCoP/NF is well matched (Tables S2–S4) with the added amounts of Ni(NO_3_)_2_ and Co(NO_3_)_2_ resources. The Al element is derived from the substrate (Al foil) during the SEM-EDS tests.

**Figure 2:**
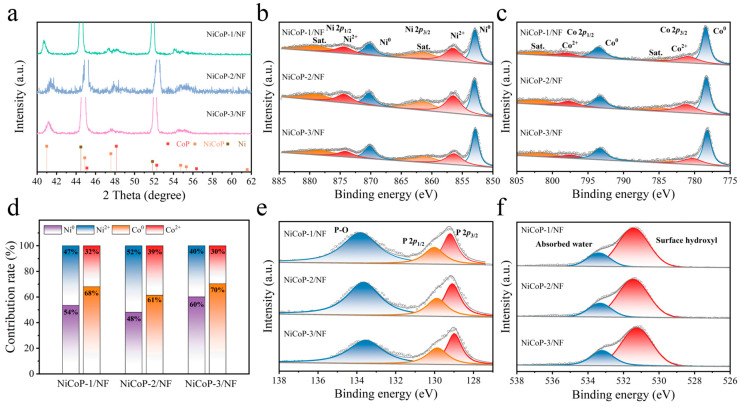
(**a**) XRD patterns of NiCoP-1/NF, NiCoP-2/NF, and NiCoP-3/NF. High-resolution XPS Ni 2p (**b**), Co 2p (**c**), P 2p (**e**), O 1s (**f**), and corresponding contents (**d**) of Ni^0^/Ni^2+^ and Co^0^/Co^2+^ sub-bands for as-prepared catalysts.

For NiCoP/NF, the diffraction peaks at 41.0°, 44.9°, 47.6°, 54.7°, 55.3°, and 61.6° can be attributed to the (111), (201), (210), (002), (211), and (301) facets of the hexagonal NiCoP (PDF No. 71-2336), whereas peaks at 45.1°, 48.1°, 52.3°, and 56.4° can be attributed to the (210), (211), (103), and (212) facets of CoP (PDF No. 29-0497) (Figure 2a). These results provide more evidence in favor of the creation of a Ni-Co-P heterogeneous structure. It can be found that NiCoP is the main crystalline phase with the adding ratio of 1:1 for Ni and Co sources, indicating that the same Ni and Co added amount can promote the formation of NiCoP.

X-ray photoelectron spectroscopy (XPS) results of NiCoP-1/NF, NiCoP-2/NF, and NiCoP-3/NF showed the presence of Co, Ni, P, and O elements (Figure S6). Figure 2b and Figure S7 show the comparisons of XPS Ni 2p spectra for NiP/NF, CoP/NF, and NiCoP/NF samples, and the corresponding sub-bands with binding energy values at about 853, 856.5, 870.3, and 874.3 eV can be attributed to Ni^0^ 2p_3/2_, Ni^2+^ 2p_3/2_, Ni^0^ 2p_1/2_, and Ni^2+^ 2p_1/2_, respectively. From the Co 2p XPS spectra in Figure 2c and Figure S8, 778.4, 781.2, 793.4, and 797.6 eV correspond to Co^0^ 2p_3/2_, Co^2+^ 2p_3/2_, Co^0^ 2p_1/2_, and Co^2+^ 2p_1/2_ [21,22,23]. Compared with NiP/NF, the increase in Co content in NiCoP/NF drives the sub-bands of Ni 2p3/2, Co 2p3/2, Ni 2p1/2, and Co 2p1/2 to move continuously to the higher binding energies, suggesting that both Co and Ni in the NiCoP samples contain partial positive charges, which promotes the adsorption of reactants. The higher oxidation states of both Ni and Co are attributed to the presence of NiO_x_-Co and/or CoO_x_-Ni metal–oxide interfaces. The transfer of electrons by oxygen between Ni and Co causes the difference in electron state density at the Ni and Co active sites at the interface, and then optimizes the adsorption energy of the hydrogen intermediate at the metal active site, consequently enhancing the HER performance [4]. At the same time, the ionicity of the M-P bond in the bimetallic phosphide increased, thus the electrons in the metal partially migrated to the phosphide [24]. Obviously, the ratios of Co^2+^ and Ni^2+^ in NiCoP-2/NF reach the maximum value, showing a strong electron transfer ability, and thus NiCoP-2/NF has the most excellent reactant adsorption activity (Figure 2d).

The peaks at element P (129.9 and 130.3 eV) are higher than the deconvolution XPS P 2p peaks at 128.3 and 130.2 eV (Figure 2e and Figure S9), suggesting that P carries a partially negative charge. At the same time, metals associated with M-PO_x_ exposed to air are likely to be in an oxidized state with a peak value of 133.4 eV [25]. The electron-rich nature of P enables M-P to act as a proton concentrator to promote the adsorption of H species on the catalyst surface. Figure 2f and Figure S10 show the comparisons of XPS O 1s spectra, and the corresponding sub-bands with binding energy values at about 531.7 and 532.9 eV can be attributed to surface hydroxyl and absorbed water, respectively [26,27]. In summary, during the HER process, P and NiCo act as the proton concentrator and H* acceptor, respectively, and after the Volmer step occurs, M-P rapidly adsorbs more H* and enhances the overall HER activity [22,28].

### 3.2. Electrocatalytic HER Performance Evaluation

**Figure 3:**
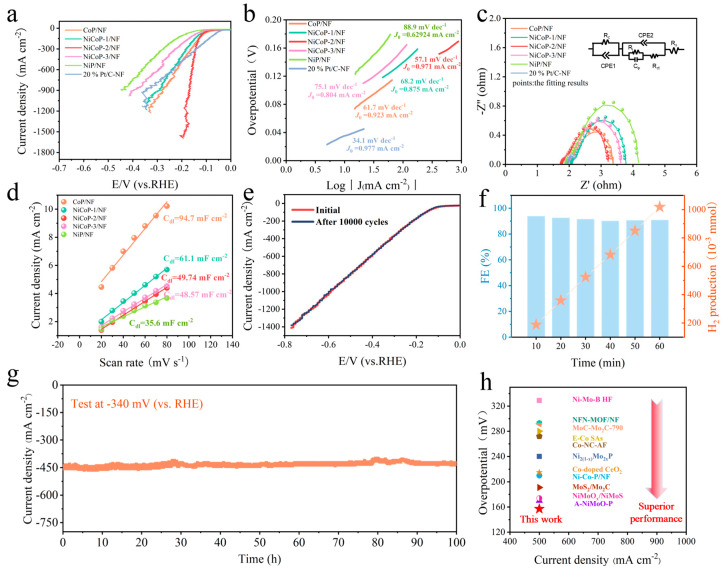
LSV curves (with 80% iR compensation) (**a**), Tafel plots (**b**), and EIS (lines and points represent the original and fitted data, respectively) (**c**) of as-prepared catalysts in 1 M KOH. (**d**) Linear dependence of capacitive current density versus scan rates. (**e**) The comparisons of LSV curves for NiCoP-2/NF before and after 10,000 test cycles. (**f**) Estimated H_2_ production and Faraday efficiency at −340 mV overpotential. (**g**) i–t curve of NiCoP-2/NF at the constant potential of −340 mV. (**h**) The comparisons of non-precious metal HER catalysts at 500 mA cm^−2^ in the literature.

Figure 3a shows that NiCoP-2/NF has the best HER activity compared to other control samples. It can be found from Figure S11 that stirring can further enhance the activity of the catalyst at high current densities, and thus it is used in our research. At overpotentials as low as 150 mV and 169 mV, NiCoP-2/NF achieves current densities as high as 500 mA cm^−2^ and 1000 mA cm^−2^, respectively, which shows its excellent HER catalytic activity. NiCoP-2/NF still exhibits the most excellent HER activity (−200 mA cm^−2^@169 mV) after the normalization of ECSA values (Table S5 and Figure S13). It is expected to be used in industrial applications. Subsequently, we used Tafel slope and electrical impedance spectroscopy (EIS) tests to examine the catalytic kinetics of HER. The Tafel slope of NiCoP-2/NF is 57.1 mV dec^−1^, which is significantly lower than those of CoP/NF (61.7 mV dec^−1^), NiCoP-1/NF (68.2 mV dec^−1^), NiCoP-3/NF (75.1 mV dec^−1^), and NiP/NF (88.9 mV dec^−1^), and slightly higher than that of 20% Pt/C-NF (34.1 mV dec^−1^) (Figure 3b). Correspondingly, NiCoP-2/NF also shows the highest exchange current density (0.971 mA cm^−2^) among as-prepared catalysts, further confirming the fastest kinetic rate. Based on these results, the rate-determined step of HER for NiCoP-2/NF could be the Heyrovsky step (that is, electrochemical desorption). The following order is shown by Figure 3c for the increase in electron transfer resistance: NiP/NF > NiCoP-1/NF > NiCoP-3/NF > NiCoP-2/NF. The catalytic activity of HER was significantly enhanced due to the reduced resistance and accelerated charge transfer/diffusion ability. The simulated equivalence circuit for NiCoP/NF is also displayed in Figure 3c, among which R_s_, R_f_, and R_ct_ are used to represent the solution resistance, high-frequency semicircle resistance, and charge-transfer resistance, respectively. In addition, CPE1 and CPE2 simulate the double-layered capacitance between the catalyst–electrode interface and electrode–electrolyte interface, respectively. Notably, the electrochemical double-layer capacitance (C_dl_) of NiCoP/NF gradually increased with the increase in Co content (Figure 3d and Figure S15), which indicated that the addition of Co element enhanced the surface roughness of the catalyst and enlarged its electrochemically active area.

After 10,000 cycles in the durability test, the LSV curves show no significant change (Figure 3e), demonstrating the excellent stability of NiCoP-2/NF. After the stability test, only a small amount of Ni and Co elements are detected in the electrolyte, while the relatively large amount of dissolved P element originated from the amorphous phosphorus oxide layer on the surface of the nanowires, which explains the origin of long-term stability for NiCoP-2/NF (Table S7). On the other hand, NiCoP-2/NF exhibited excellent electrocatalytic durability for HER and was able to maintain a very stable state for up to 100 h at an industrial current density of about 450 mA cm^−2^ (Figure 3g). In addition, the components of NiCoP-2/NF did not change significantly after the 100 h test period from XRD (Figure S16), while NiCoP-2/NF still maintained good nanowire morphology (Figure S17). EDS mapping measurement also showed an almost unchanged distribution of Ni, Co, P, and O elements (Figure S18), suggesting that NiCoP-2/NF has good chemical and structural stability at high current densities. The H_2_ produced during the reaction was collected using the drainage gas collection method and compared to the theoretical gas: HER Faraday efficiency (FE%) of NiCoP-2/NF was found to be higher than 90% (Figure 3f). A comparison between Figure 3h and Table S8 shows that the HER performance of NiCoP-2/NF is superior to most of the catalysts found so far, suggesting that it has the capacity for large-scale H_2_ production.

### 3.3. Insight into the HER Mechanism

**Figure 4:**
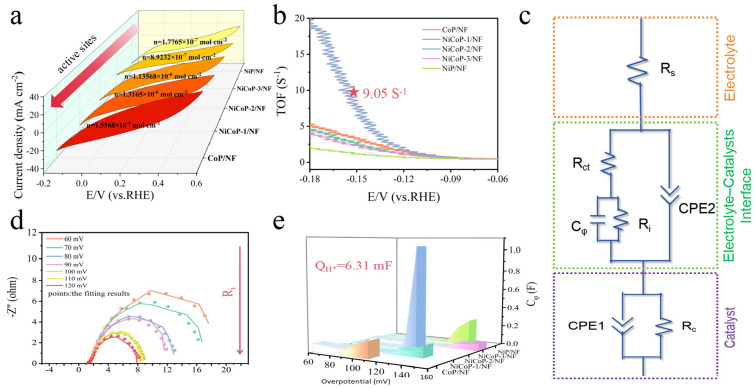
(**a**) Number of active sites and (**b**) TOFs for different catalysts. (**c**) Schematic diagram of the electrode structure and the equivalent circuit model. (**d**) Nyquist plots of NiCoP-2/NF (lines and points represent the original and fitted data, respectively) and (**e**) Fitted data of C_φ_ for as-prepared catalysts at varied overpotentials during HER.

To investigate the causes of the improved HER performance of NiCoP/NF in more detail, the active site densities of different catalysts and the TOF (turnover frequency) of each site were determined by electrochemical methods [14,29]. Furthermore, after considering the effect of different mass loadings on the catalytic activity, the C_dl_ values of as-prepared catalysts through the normalization of mass activity were also displayed in Table S6. All samples did not show any obvious redox peaks in the electrolyte solution (pH = 7) at the set voltage range, so it can be assumed that the active site density of the catalysts is linearly related to the area of the integral redox peaks. Figure 4a shows that the active site densities of these as-prepared catalysts increase gradually with the increase in Co content, proving that the introduction of Co in NiCoP can increase the electrochemically active area. It is noteworthy that the electrochemical performance of these catalysts did not show a linear enhancement like the active site density. Attributed to the synergistic catalytic effect of Co and Ni, it is reasonable to believe that the addition of Ni into the catalyst increases its intrinsic activity compared to monometallic phosphides (CoP/NF). At −150 mV of overpotential (Figure 4b), NiCoP-2/NF has better TOF (9.05 S^−1^) than NiP/NF (1.18 S^−1^) and CoP/NF (3.34 S^−1^).

In addition, the adsorption of H* on NiCoP-2/NF was further analyzed by microkinetics. Simulations were carried out using an equivalent circuit model [5] based on the obtained EIS data (Figure 4c). The adsorption resistance in terms of R_i_ in the second parallel component can indicate the hydrogen adsorption of catalyst on the surface. The C_φ_ is the hydrogen adsorption pseudocapacitance, and the hydrogen adsorption charge (Q_H*_) can be calculated by integrating with the overpotential. At lower voltages, the values of C_φ_ obtained are smaller and voltage-independent, while R_i_ is larger, indicating negligible hydrogen adsorption. As the voltage increased, R_i_ gradually decreased (Figure 4d and Figure S19), which promoted the hydrogen adsorption on the catalyst surface. When the voltage was further increased, accompanied by the acceleration of the kinetic rate of hydrogen adsorption, Q_H*_ reached the maximum value, indicating that the catalyst surface reached the saturation of hydrogen adsorption [30]. In addition, as the voltage increases, the semicircle in the plot gradually shrinks until it closes, which represents that the H* coverage on the surface of NiCoP-2/NF reaches the maximum. The integration results show that NiCoP-2/NF exhibits the most hydrogen adsorption with a charge of 6.31 mF at an overpotential of 120 mV (Figure 4e and Table S9). Thus, the outstanding HER performance of NiCoP-2/NF is a result of the synergistic catalytic effect between nickel and cobalt, whereby Ni enhances the intrinsic activity of NiCoP, and Co expands the electrochemically active area of the catalyst during the catalyst fabrication process.

### 3.4. Evaluation of the Overall Water Splitting

**Figure 5:**
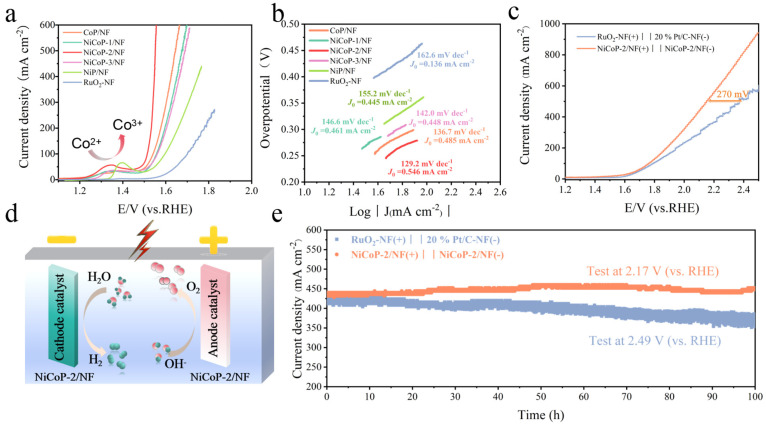
LSV curves (with 80% iR correction) (**a**) and Tafel plots (**b**) of as-prepared catalysts for OER by using Hg/HgO as reference electrode. (**c**) Polarization curves of NiCoP-2/NF||NiCoP-2/NF and Pt/C/NF||RuO_2_-NF toward overall water splitting in 1 M KOH. (**d**) A two-electrode membrane-free electrolyser for hydrogen production from water splitting. (**e**) Time-dependent current density curves for NiCoP-2/NF||NiCoP-2/NF and 20% Pt/C-NF||RuO_2_-NF at constant potentials of 2.17 and 2.49 V (the current density keeps ~450 mA cm^−2^) for 100 h.

NiCoP-2/NF had notably superior onset potential and overpotentials during the OER process compared to the other samples, with a voltage of 1.56 V at the current density of 500 mA cm^−2^ (Figure 5a). In addition, NiCoP-2/NF showed a clear redox peak at 1.3 V, indicating the presence of Co^2+^ → Co^3+^ [31]. Compared to CoP/NF (136.7 mV dec^−1^), NiCoP-1/NF (146.6 mV dec^−1^), NiCoP-3/NF (142.0 mV dec^−1^), NiP/NF (155.2 mV dec^−1^), and RuO_2_-NF (162.6 mV dec^−1^), NiCoP-2/NF shows a smaller Tafel slope of 129.2 mV dec^−1^, indicating good catalytic kinetics (Figure 5b). Meanwhile, NiCoP-2/NF also shows the highest exchange current density value (0.546 mA cm^−2^) among the as-prepared catalysts, further confirming its fastest kinetic rate. Furthermore, the Nyquist plots for varied catalysts (including commercial RuO_2_-NF) under OER conditions are shown in Figure S22, among which NiCoP-2/NF also displays the lowest electron transfer resistance. Calculations of mass activity indicate that NiCoP-2/NF has excellent catalytic activity in both OER and HER processes (Figures S14 and S23). In order to extend the scope of this study and evaluate the performance of NiCoP-2/NF in a more realistic situation (Figure 5d), the overall water splitting system was constructed by using NiCoP-2/NF as both cathode and anode catalysts, simultaneously. Compared to 20% Pt/C-NF||RuO_2_-NF (1.78 V@100 mA cm^−2^, 2.40 V@500 mA cm^−2^), the NiCoP-2/NF||NiCoP-2/NF system can drive current densities of 100 mA cm^−2^ and 500 mA cm^−2^ at cell voltages as low as 1.74 V and 2.13 V (Figure 5c). Importantly, NiCoP-2/NF||NiCoP-2/NF also exhibits good stability up to 100 h (~450 mA cm^−2^) (Figure 5e). The test findings indicate that NiCoP-2/NF||NiCoP-2/NF has the higher activity and durability in water electrolysis compared to 20% Pt/C-NF||RuO_2_-NF, which provides good potential for industrial applications.

## 4. Conclusions

In this work, a bimetallic NiCoP phosphide has been constructed on NF as an efficient HER catalyst through a two-step method. The optimal NiCoP-2/NF catalyst requires the rather low overpotentials of 150 mV and 169 mV to reach industrial-scale current densities (500 and 1000 mA cm^−2^), surpassing the majority of reported non-precious metal catalysts. In the meantime, the NiCoP-2/NF catalyst demonstrates high Faradaic efficiency over 90% and good durability of 100 h at a current density of ~450 mA cm^−2^. Based on the above studies, Ni improves the intrinsic activity of a single active site, and the introduction of Co increases the amount of active sites, thus resulting in excellent HER activity. In the meantime, the good stability of the as-prepared NiCoP-2/NF is attributed as the reason for the protection of the externally generated amorphous phosphorus oxide layer. In addition, the overall water splitting electrolyzer established by using NiCoP-2/NF as both an HER and OER catalyst has an output current density of 500 mA cm^−2^ at 2.13 V, which is ~270 mV lower than that of 20% Pt/C-NF||RuO_2_-NF. The construction of bimetallic phosphide in this study can provide a new perspective on the alkaline electrolysis of water for hydrogen production under a high current density.

## Data Availability

Data are provided if readers request it.

## Supplementary Materials

The following supporting information can be downloaded at: https://www.mdpi.com/article/10.3390/nano14100853/s1, Figure S1. Typical SEM images at different magnifications of NiCoP-1/NF; Figure S2. Typical SEM images at different magnifications of NiCoP-3/NF; Figure S3. Typical SEM images at different magnifications of NiP/NF; Figure S4. Typical SEM images at different magnifications of CoP/NF; Figure S5. SEM-EDS spectra of NiCoP-1/NF, NiCoP-2/NF and NiCoP-3/NF. (The Al signal is derived from the substrate (Al foil) during the testing process); Figure S6. Survey scan of XPS spectra of the NiCoP-1/NF, NiCoP-2/NF and NiCoP-3/NF; Figure S7. High-resolution XPS Ni 2p of CoP/NF and NiP/NF; Figure S8. High-resolution XPS Co 2p of CoP/NF; Figure S9. High-resolution XPS P 2p of CoP/NF and NiP/NF; Figure S10. High-resolution XPS O 1s of NiP/NF and CoP/NF; Figure S11. LSV curves of as-prepared catalysts with/without stirring in HER; Figure S12. The LSV curves without iR compensation in 1 M KOH; Figure S13. LSV curves after normalization of ECSA values; Figure S14. The mass activity curves of as-prepared catalysts for HER; Figure S15. The CV curves of varied samples with the scan rate ranging from 20 to 80 mV s^−1^ in 1.0 M KOH; (a) CoP/NF; (b) NiCoP-1/NF; (c) NiCoP-2/NF; (d) NiCoP-3/NF; (e) NiP/NF; Figure S16. XRD patterns for NiCoP-2/NF after the stability tests; Figure S17. SEM image of NiCoP-2/NF after the stability tests; Figure S18. TEM image and EDS elemental mappings of NiCoP-2/NF after the stability tests; Figure S19. Nyquist plots of (a) CoP/NF, (b) NiCoP-1/NF, (c) NiCoP-3/NF and (d) NiP/NF for HER at varied potentials (lines and points represent the original data and fitted data, respectively); Figure S20. LSV curves of as-prepared catalysts with/without stirring in OER; Figure S21. LSV curves (without iR correction) of as-prepared catalysts for OER by using Hg/HgO as reference electrode; Figure S22. The Nyquist plots of as-prepared catalysts and commercial RuO_2_-NF measured in OER conditions (lines and points represent the original and fitted data, respectively); Figure S23. The mass activity curves of as-prepared catalysts for OER; Table S1. Mass loading during the preparation of different catalysts; Table S2. The corresponding mass and atomic percentages in SEM-EDS spectra of NiCoP-1/NF, NiCoP-2/NF and NiCoP-3/NF; Table S3. Corresponding mass percentages and atomic percentages in TEM-EDS images of NiCoP-2/NF; Table S4. ICP data of Ni-Co-Pre/NF; Table S5. ECSA values of as-prepared catalysts; Table S6. C_dl_ values of as-prepared catalysts through the normalization of current density and mass activity, respectively; Table S7. ICP data of NiCoP-2/NF solution after 10,000 cycles of testing; Table S8. Comparison of electrocatalytic activities of HER in 1.0 M KOH between NiCoP-2/NF in this work and various transition metal based catalysts recently reported; Table S9. The fitted parameters of the EIS data of various NiCoP/NF catalysts. Refs [32,33,34,35,36,37,38,39,40,41,42] are cited in Supplementary Materials.
