# Supplementary material for: Synergetic Catalytic Effect between Ni and Co in Bimetallic Phosphide Boosting Hydrogen Evolution Reaction
Source: Nanomaterials (Basel). 2024 May 14;14(10):853. doi: 10.3390/nano14100853 (PMC11123732; doi:10.3390/nano14100853)
Supplement: Supplementary file 1 [file nanomaterials-14-00853-s001.zip › nanomaterials-2949396-supplementary.pdf]

Synergetic catalytic effect between Ni and Co in bimetallic phosphide boosting hydrogen evolution reaction

Xiaohan Wang # [1,2,3], Han Tian # [1], Libo Zhu [1,3], Shujing Li [2], Xiangzhi Cui [1,2,3,*]

[1] Shanghai Institute of Ceramics, Chinese Academy of Sciences, Shanghai 200050, P. R. China.
[2] School of Chemistry and Materials Science, Hangzhou Institute for Advanced Study, University of Chinese Academy of Sciences, Hangzhou 310024, P. R. China.
[3] Center of Materials Science and Optoelectronics Engineering, University of Chinese Academy of Sciences, Beijing 100049, P. R. China.
* Correspondence: cuixz@mail.sic.ac.cn (X.C.)
# These authors contribute equally.

EXPERIMENTAL SECTION

Materials Characterization

The measurements were done using a field emission scanning electron microscope (SEM) (FEI Magellan-400, 5 kV). With a 200 kV JEOL JEM-F200 transmission electron microscope using a field-emission electron gun, the transmission electron microscopic (TEM), high-resolution TEM (HRTEM), and associated energy dispersive X-ray spectrometer mapping (EDS-mapping) images were obtained. The Cu K_α radiation target (40 kV, 40 mA) was used to get the X-ray diffraction (XRD) patterns from the Rigaku D/Max-2550V X-ray diffractometer at a scan rate of 4 ° min^{-1}. Thermo Fisher Scientific ECSA lab 250 XPS spectrometer was used to detect the X-ray photoelectron spectroscope (XPS) signals using monochromatic Al Kα X-rays. The carbon base (284.8 eV) was used for calibration. Optical emission spectroscopy (ICP-OES, Agilent 725) using inductively coupled plasma was used to assess the catalyst's composition and concentration.

HER products analysis

The Faraday efficiency of the catalyst to produce hydrogen was evaluated using the following equation

$$\mathrm{FE}(\%) = \frac{\mathrm{n_e} \times \mathrm{V_{(H_2 production)}} \times \mathrm{F}}{\mathrm{Q_{tatal}} \times \mathrm{V_s}} \times 100\%$$

n_e is the number of electrons required to produce one molecule of hydrogen (n_e = 2), F is Faraday constant (96485 C mol^{-1}), Q_{total} is the total number of electrons during the reaction, and Vs is the standard molar volume (22.4 L mol^{-1}).

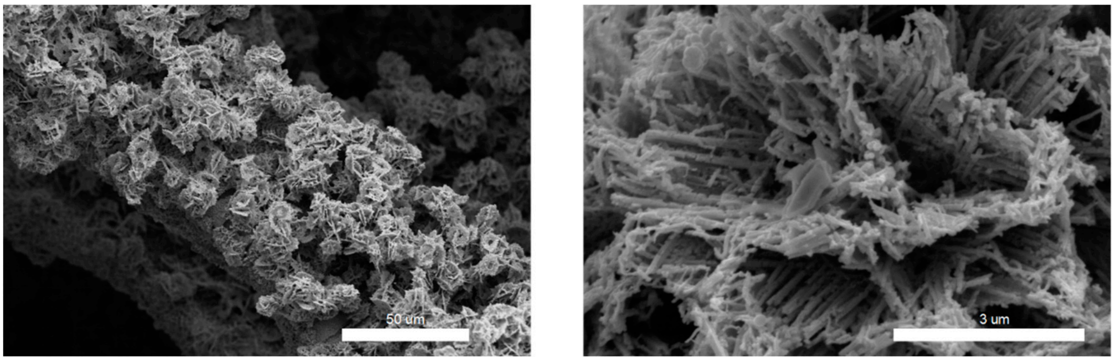

Figure S1. Typical SEM images at different magnifications of NiCoP-1/NF.

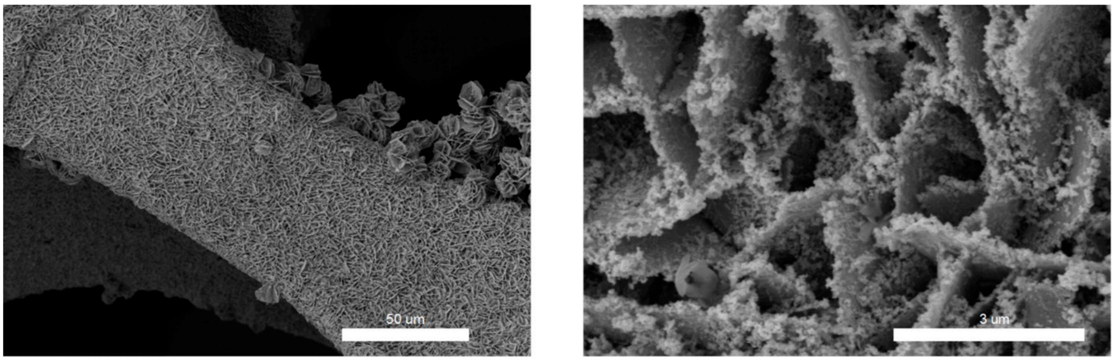

Figure S2. Typical SEM images at different magnifications of NiCoP-3/NF.

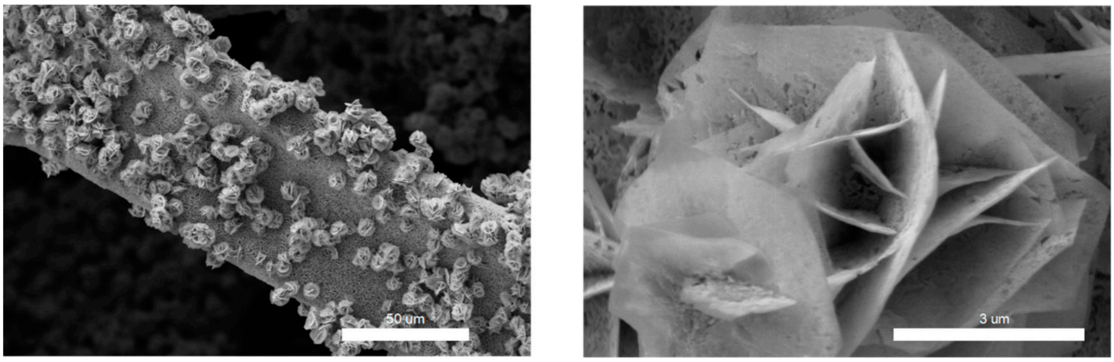

Figure S3. Typical SEM images at different magnifications of NiP/NF.

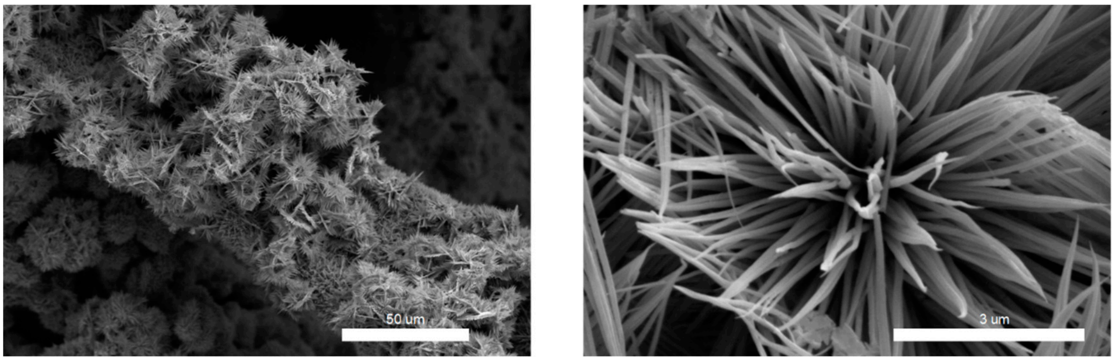

Figure S4. Typical SEM images at different magnifications of CoP/NF.

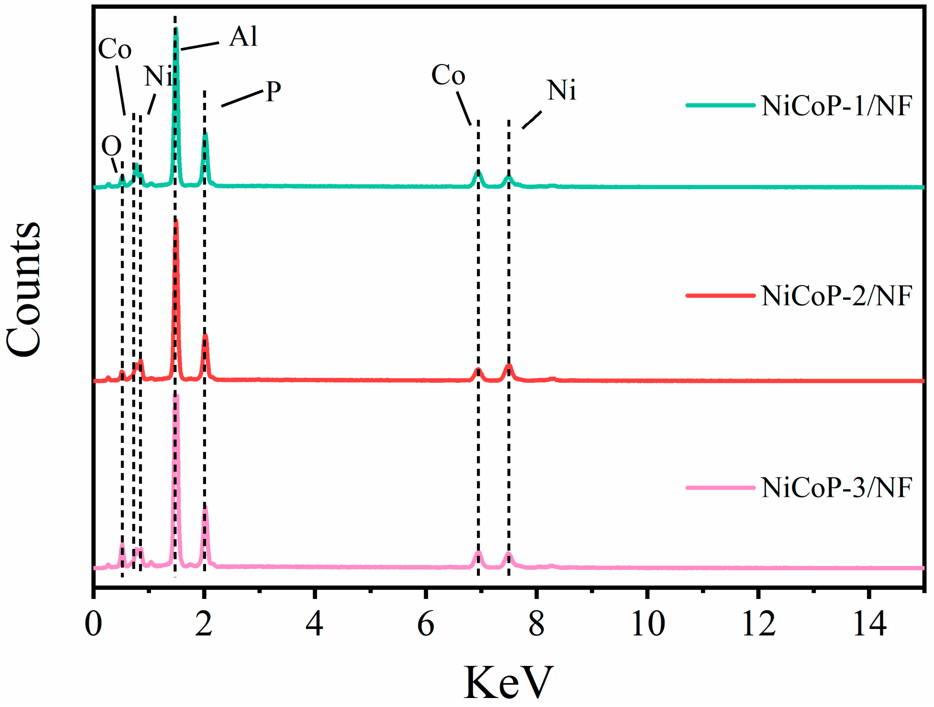

Figure S5. SEM-EDS spectra of NiCoP-1/NF, NiCoP-2/NF and NiCoP-3/NF. (The Al signal is derived from the substrate (Al foil) during the testing process)

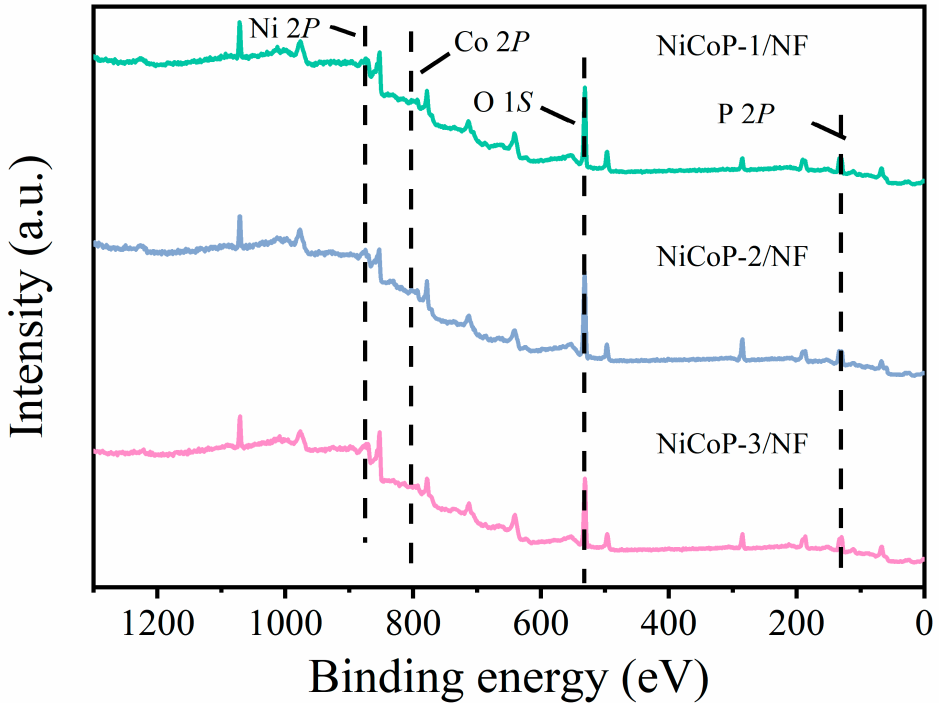

Figure S6. Survey scan of XPS spectra of the NiCoP-1/NF, NiCoP-2/NF and NiCoP-3/NF.

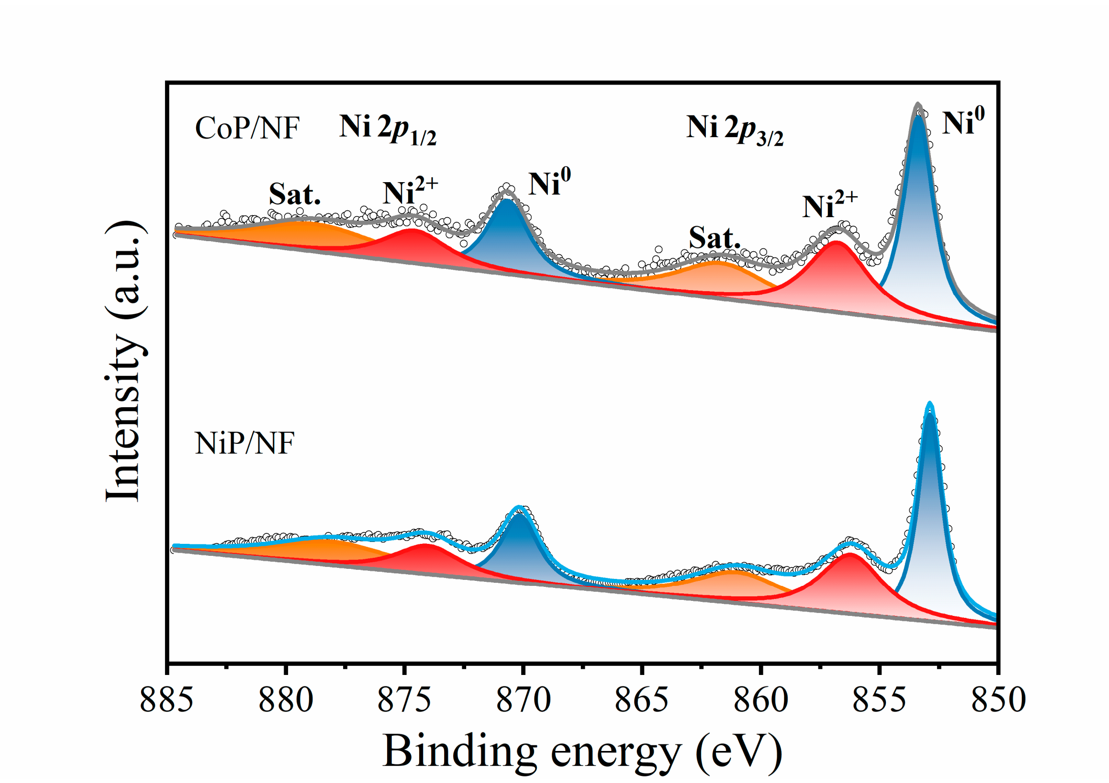

Figure S7. High-resolution XPS Ni 2p of CoP/NF and NiP/NF.

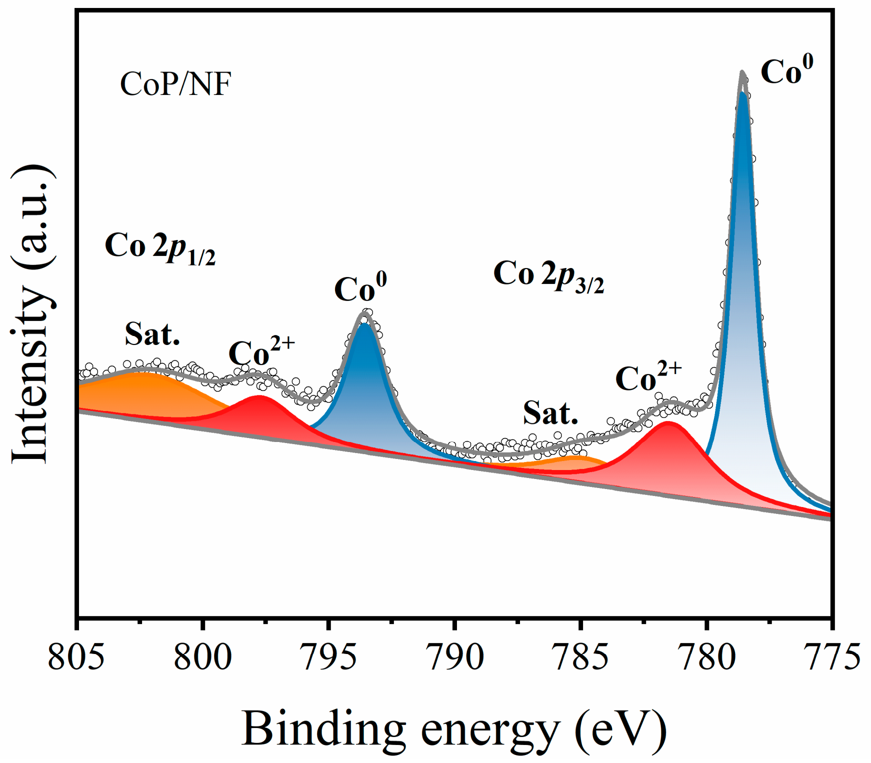

Figure S8. High-resolution XPS Co 2p of CoP/NF.

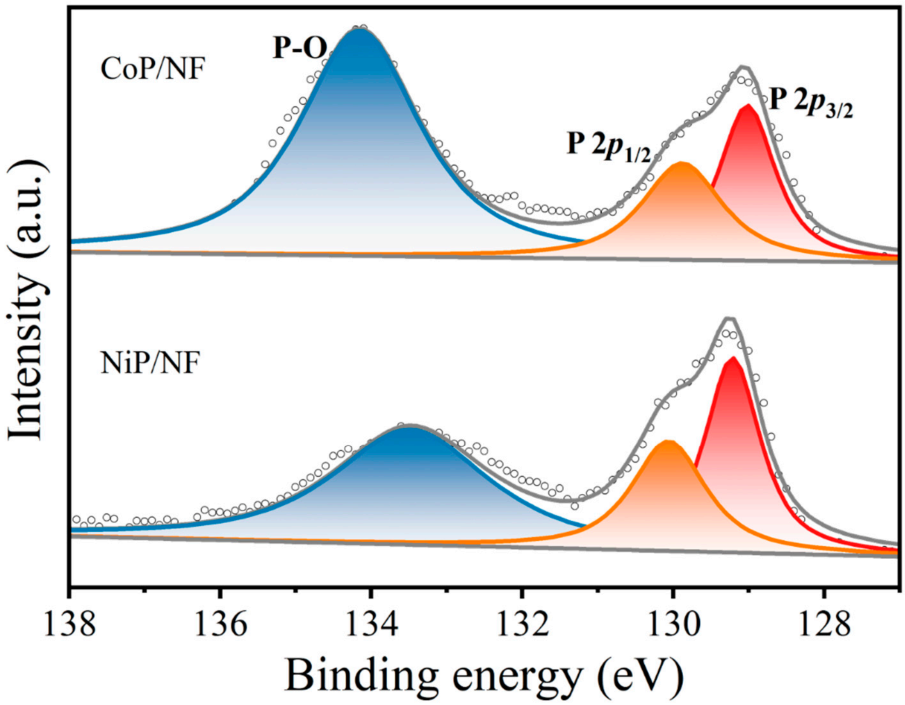

Figure S9. High-resolution XPS P 2p of CoP/NF and NiP/NF.

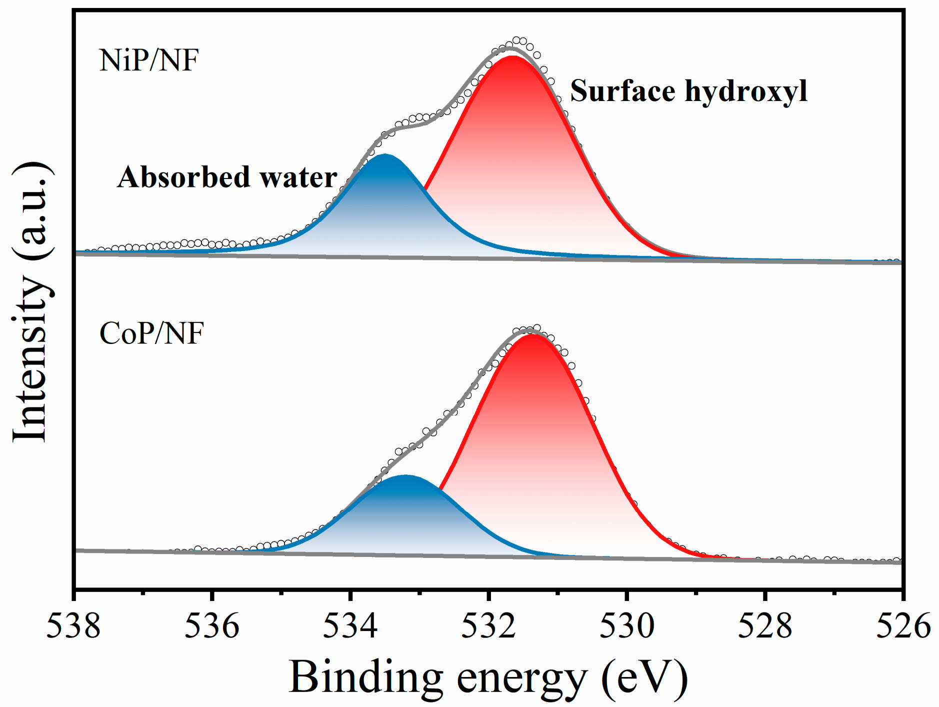

Figure S10. High-resolution XPS O 1s of NiP/NF and CoP/NF.

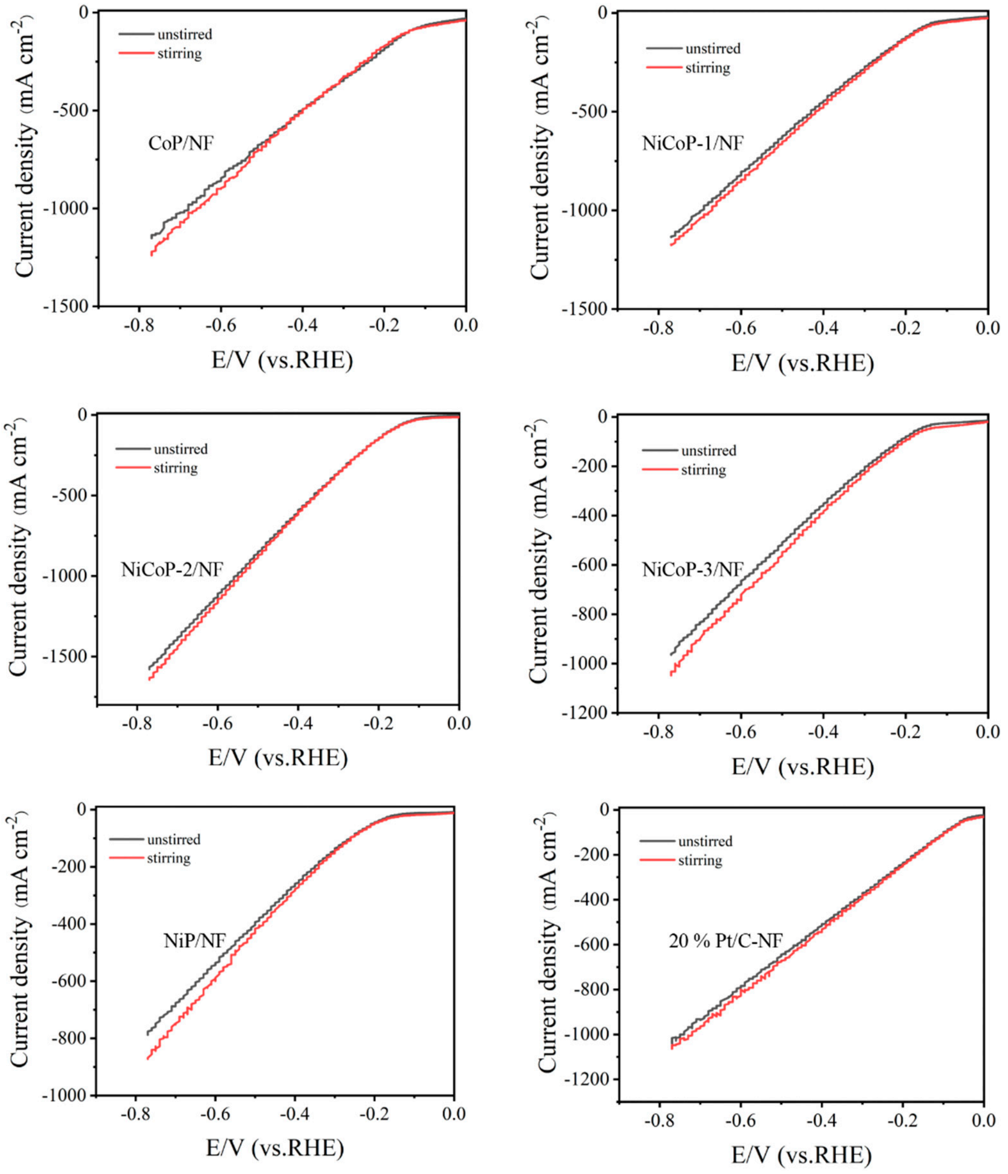

Figure S11. LSV curves of as-prepared catalysts with/without stirring in HER.

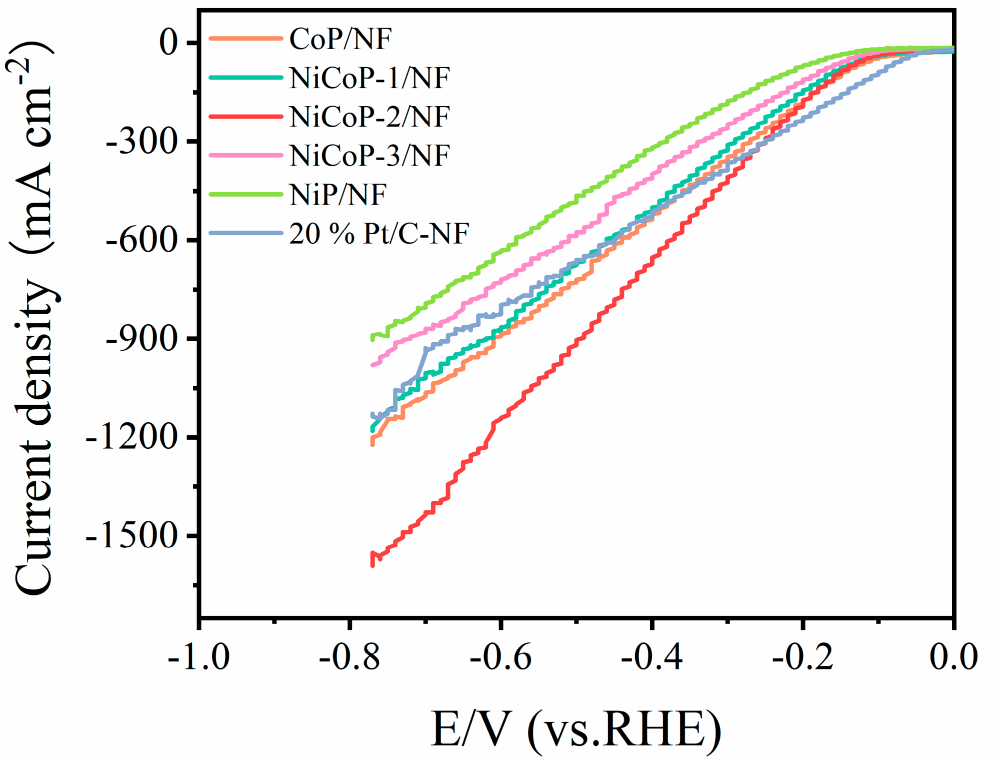

Figure S12. The LSV curves without iR compensation in 1 M KOH.

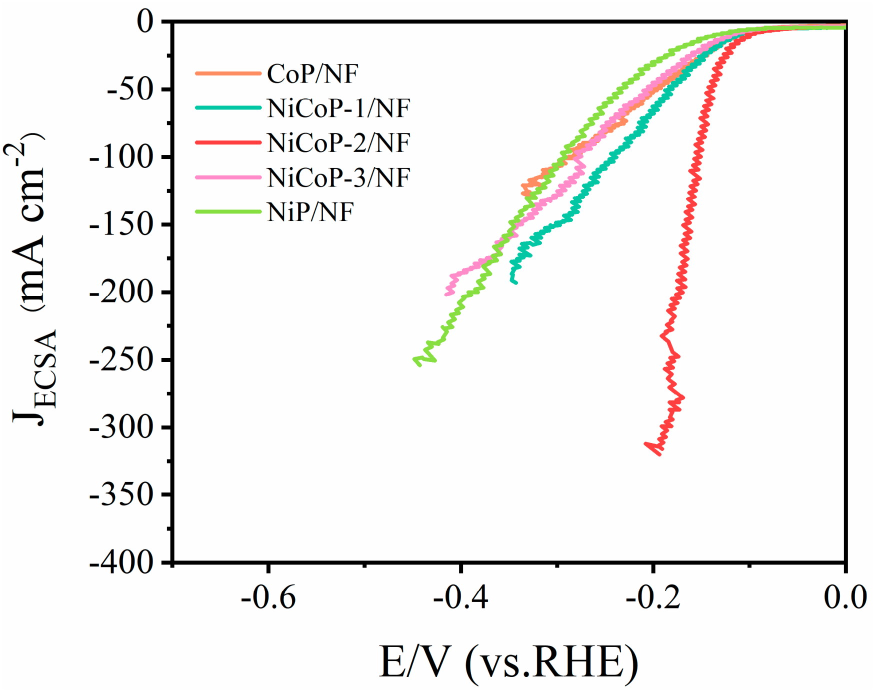

Figure S13. LSV curves after normalization of ECSA values.

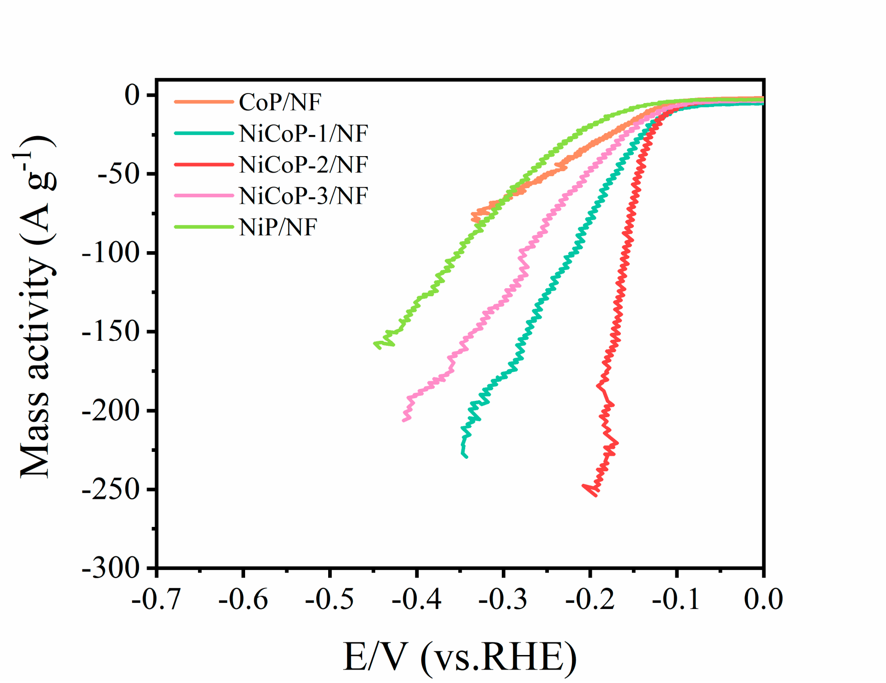

Figure S14. The mass activity curves of as-prepared catalysts for HER.

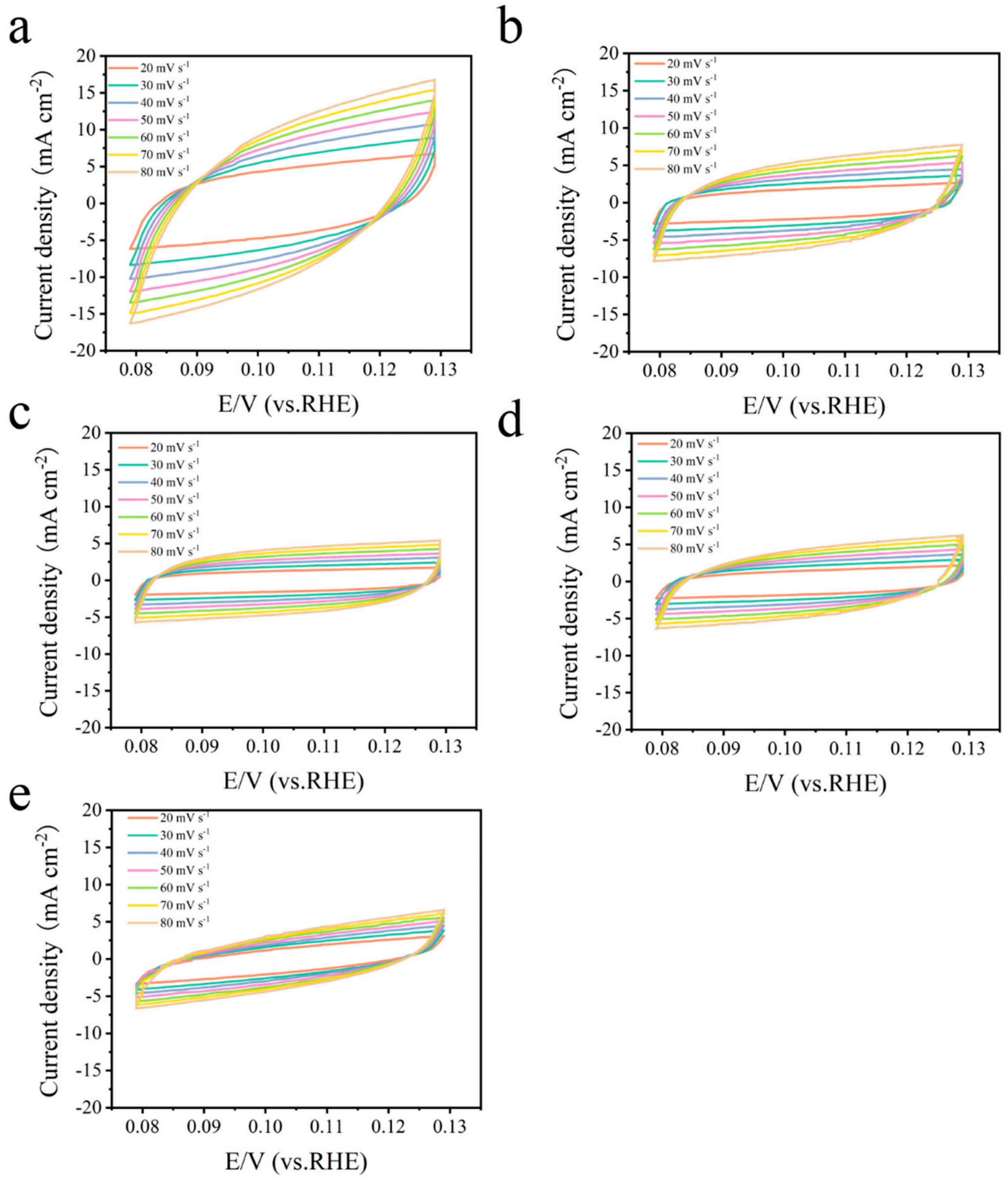

Figure S15. The CV curves of varied samples with the scan rate ranging from 20 to 80 mV s^{-1} in 1.0 M KOH; (a) CoP/NF; (b) NiCoP-1/NF; (c) NiCoP-2/NF; (d) NiCoP-3/NF; (e) NiP/NF.

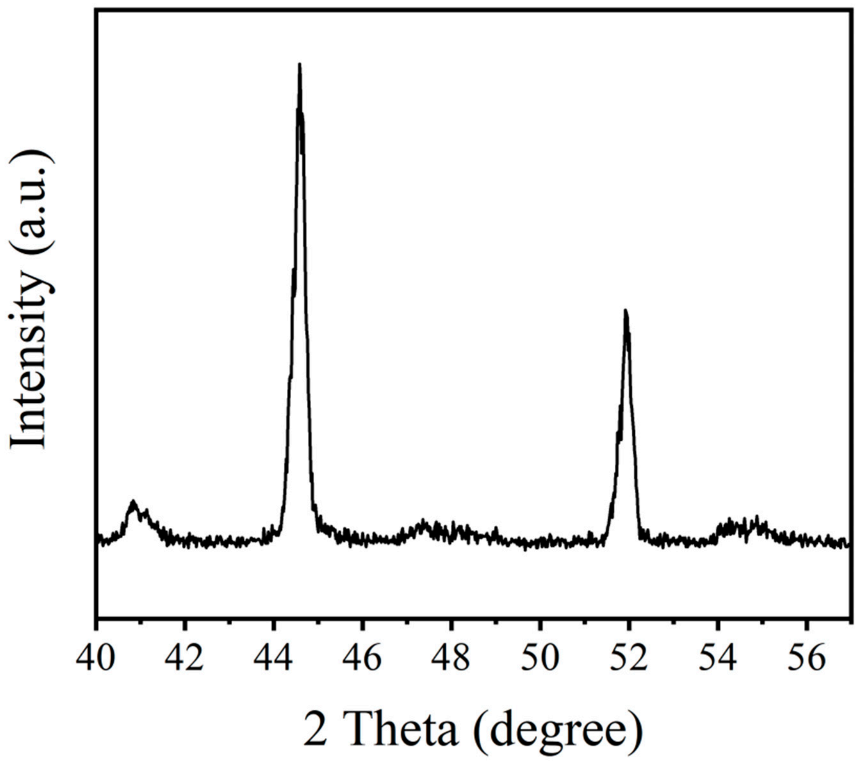

Figure S16. XRD patterns for NiCoP-2/NF after the stability tests.

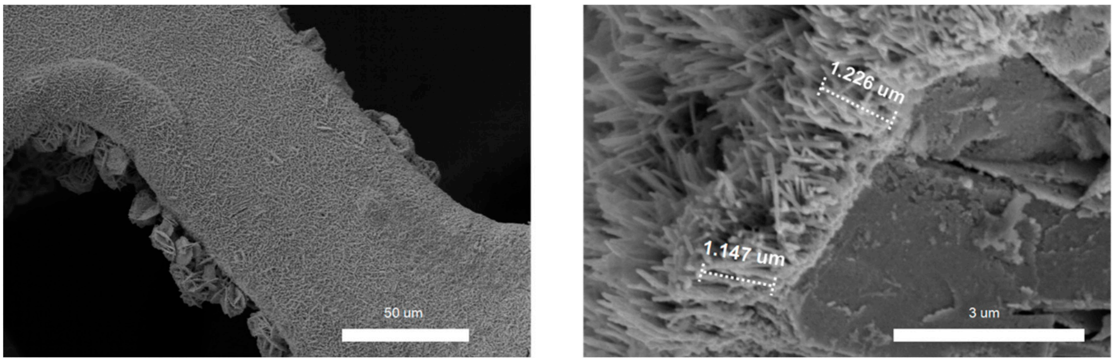

Figure S17. SEM image of NiCoP-2/NF after the stability tests.

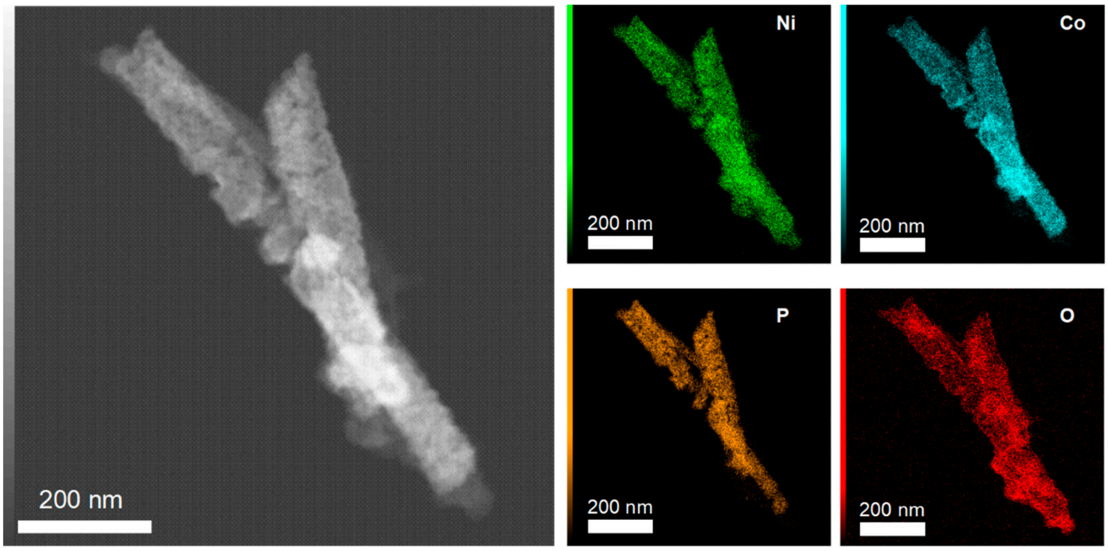

Figure S18. TEM image and EDS elemental mappings of NiCoP-2/NF after the stability tests.

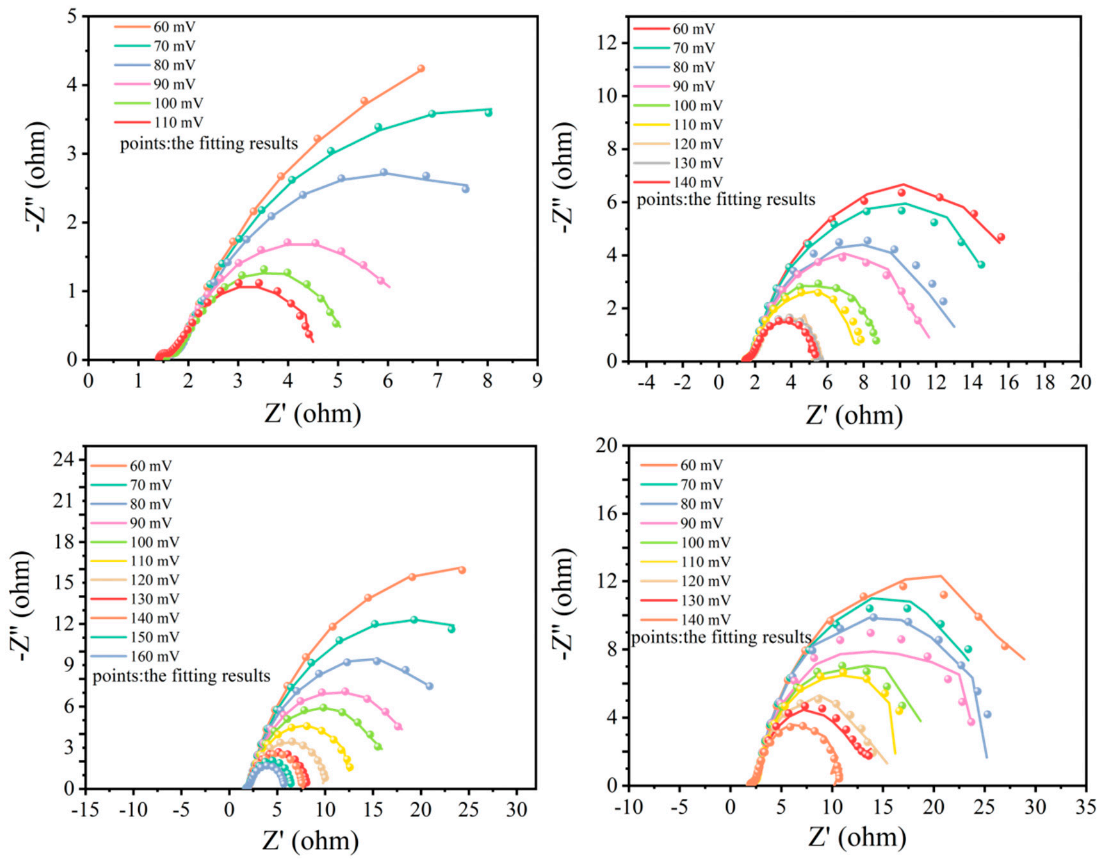

Figure S19. Nyquist plots of (a) CoP/NF, (b) NiCoP-1/NF, (c) NiCoP-3/NF and (d) NiP/NF for HER at varied potentials (lines and points represent the original data and fitted data, respectively).

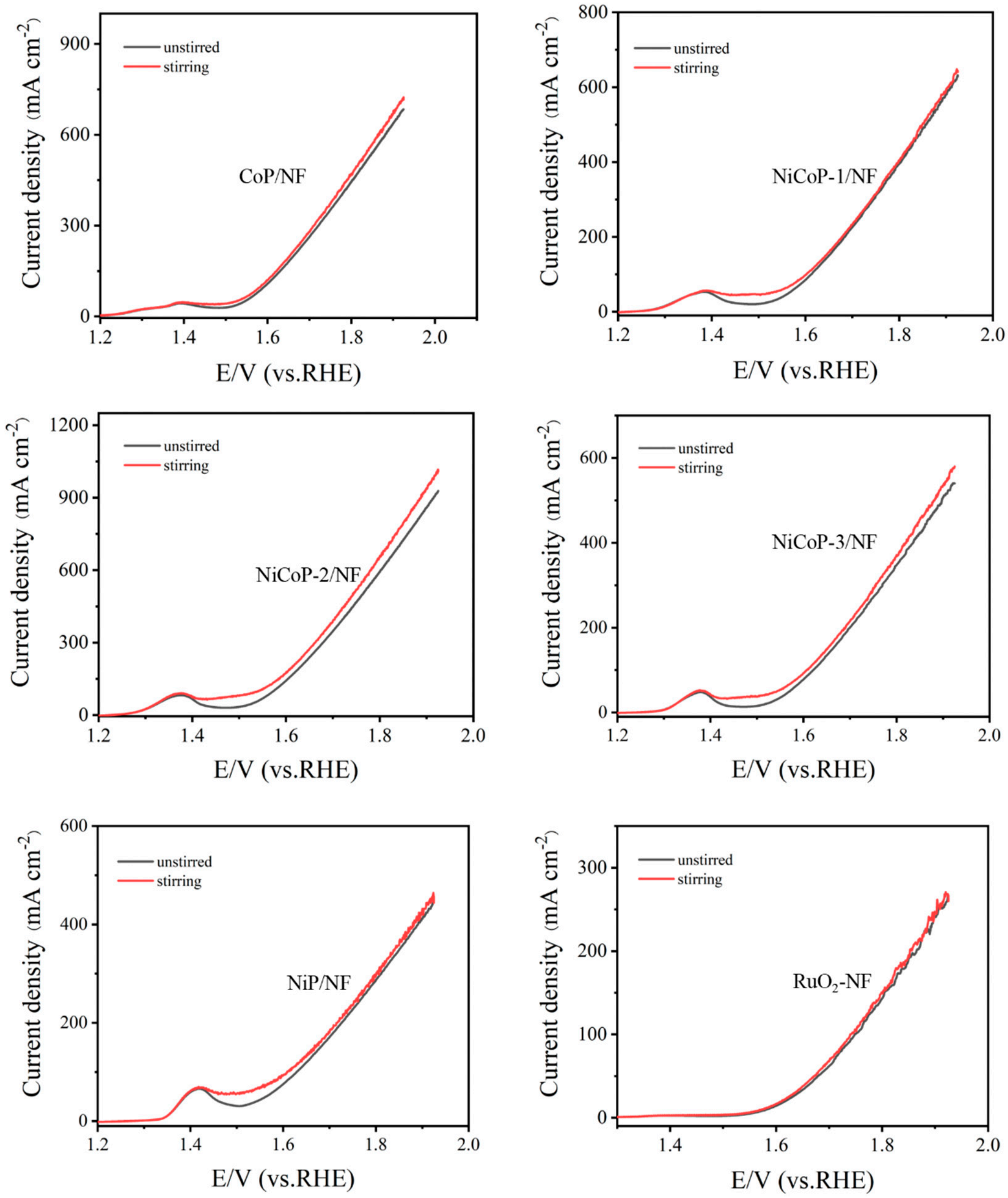

Figure S20. LSV curves of as-prepared catalysts with/without stirring in OER.

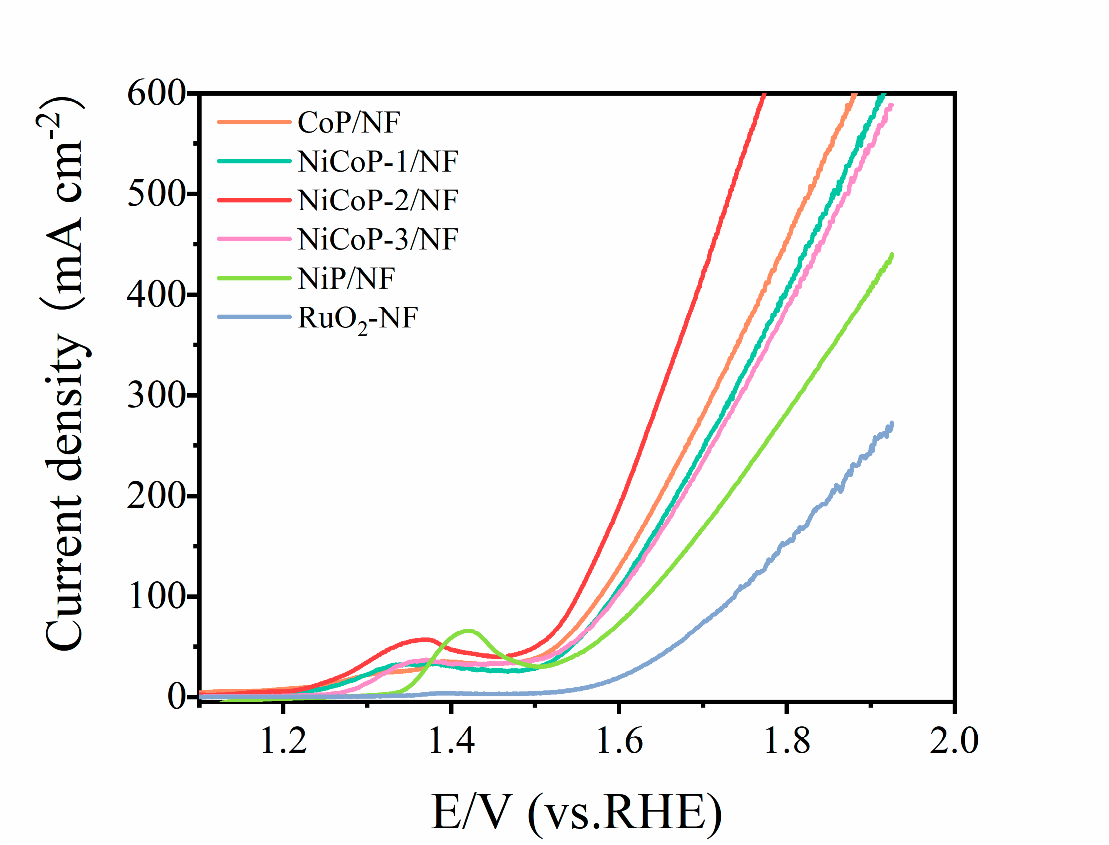

Figure S21. LSV curves (without iR correction) of as-prepared catalysts for OER by using Hg/HgO as reference electrode.

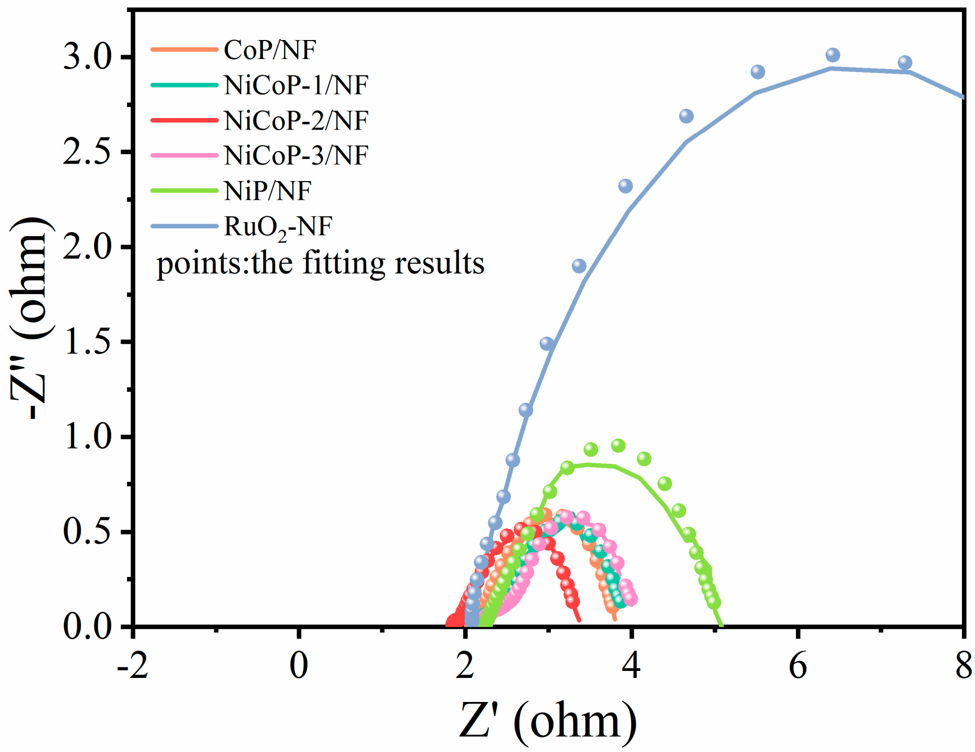

Figure S22. The Nyquist plots of as-prepared catalysts and commercial RuO_2-NF measured in OER conditions (lines and points represent the original and fitted data, respectively).

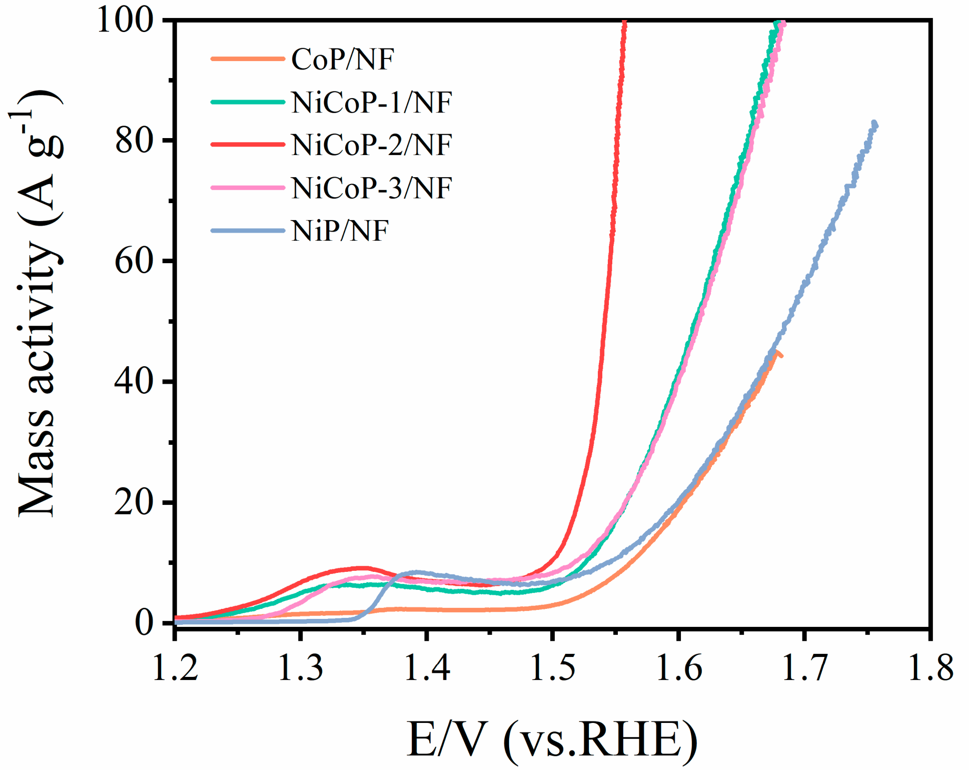

Figure S23. The mass activity curves of as-prepared catalysts for OER.

Table S1. Mass loading during the preparation of different catalysts.

Samples	Weight of substrate (NF)	Weight after preparation	Mass loading
CoP/NF	0.2081 g	0.3145 g	15.2 mg cm^{-2}
NiCoP-1/NF	0.2031 g	0.2391 g	5.143 mg cm^{-2}
NiCoP-2/NF	0.2033 g	0.2472 g	6.271 mg cm^{-2}
NiCoP-3/NF	0.2047 g	0.2380 g	4.757 mg cm^{-2}
NiP/NF	0.2009 g	0.2403 g	5.629 mg cm^{-2}
20 % Pt/C-NF	-	-	1 mg cm^{-2}
RuO_2-NF	-	-	1 mg cm^{-2}

Table S2. The corresponding mass and atomic percentages in SEM-EDS spectra of NiCoP-1/NF, NiCoP-2/NF and NiCoP-3/NF.

Samples	Element	Weight%	Atom%
NiCoP-1/NF	O	6.9	14.0
	Al	39.0	46.7
	P	19.7	20.5
	Co	19.2	10.5
	Ni	15.1	8.3
NiCoP-2/NF	O	12.5	23.7
	Al	36.9	41.7
	P	18.1	17.8
	Co	15.8	8.2
	Ni	16.7	8.7
NiCoP-3/NF	O	6.3	13.0
	Al	39.6	48.2
	P	17.1	18.1
	Co	14.0	7.8
	Ni	22.9	12.8

Table S3. Corresponding mass percentages and atomic percentages in TEM-EDS images of NiCoP-2/NF.

Element	Mass%	Atom%
C	87.66	95.07
O	2.12	1.72
P	4.03	1.69
Co	3.06	0.68
Ni	2.54	0.56

Table S4. ICP data of Ni-Co-Pre/NF.

**Element**	Ni	Co
**Weight Percentage**	25.4 wt. %	24.5 wt.%
**ICP Content**	253.83 g kg^{-1}	244.79 g kg^{-1}

Table S5. ECSA values of as-prepared catalysts.

**Samples**	**ECSA (cm^2)**
CoP/NF	2.37
NiCoP-1/NF	1.53
NiCoP-2/NF	1.24
NiCoP-3/NF	1.21
NiP/NF	0.89

Table S6. C_{dl} values of as-prepared catalysts through the normalization of current density and mass activity, respectively.

**Samples**	**mF cm^{-2}**	**mF/g**
CoP/NF	94.7	6230.3
NiCoP-1/NF	61.1	11880.2
NiCoP-2/NF	49.74	7931.8
NiCoP-3/NF	48.57	10210.2
NiP/NF	35.6	6324.4

Table S7. ICP data of NiCoP-2/NF solution after 10000 cycles of testing.

**Element**	Ni	Co	P
**Weight Percentage**	0.0068 wt. %	0.035 wt. %	95.8 wt. %
**ICP Content**	15.0 ug L^{-1}	76.8 ug L^{-1}	2073.7 ug L^{-1}

Table S8. Comparison of electrocatalytic activities of HER in 1.0 M KOH between NiCoP-2/NF in this work and various transition metal based catalysts recently reported.

Materials	Current density (mA cm^{-2})	Potential (mV vs. RHE)	Tafel slope (mV dec-1)	Stability (h)	Reference
NiCoP-2/NF	100<br>500<br>1000	122<br>150<br>169	51.6	100	This work
$Ni_{2(1-x)}Mo_{2x}P$	500	240	46.4	160	[1]
Ni-Co-P/NF	500	210	46	24	[2]
A-NiMoO-P	500	170	87	1000	[3]
$NiMoO_x$/ NiMoS	500	174	38	50	[4]
MoC-Mo_2C-790	500	292	59	1000	[5]
MoS_2/Mo_2C	500	191	44	24	[6]
Co-doped CeO_2	500	215	103.8	14	[7]
Co-NC-AF	500	272	67.6	32	[8]
NFN-MOF/NF	500	293	35.2	30	[9]
E-Co SAs	500	280	105	500	[10]
Ni-Mo-B HF	500	329	58.7	300	[11]

Table S9. The fitted parameters of the EIS data of various NiCoP/NF catalysts.

Samples	η(mV)	R_s	CPE_1	R_{ct}	$C_φ$(F)	R_i	CPE_2	R_c
CoP/NF	60	1.522	0.002681	0.1813	0.08815	15.19	0.0004005	1.033
	70	1.54	0.002859	0.1938	0.07922	11.4	0.0003987	11.07
	80	1.561	0.003031	20.03	0.07143	8.403	0.0003769	0.1183
	90	1.509	0.004856	0.2321	0.07026	4.813	0.0004741	1.372
	100	1.552	0.005458	0.166	0.1368	1.814	0.09711	1.731
	110	1.412	0.0002759	15.95	0.1752	1.401	0.07525	1.698
NiCoP-1/NF	60	1.468	0.00133	0.3313	0.02364	17	0.0002515	0.0143
	70	1.455	0.01786	0.3479	0.02154	14.74	0.0003675	0.1483
	80	1.447	0.001797	0.3739	0.01998	11.49	0.0002763	1.768
	90	1.504	0.002	0.407	0.0183	9.552	0.0003439	0.0172
	100	1.442	0.00153	4.031	0.01712	6.938	0.0002953	0.1557
	110	1.446	0.001962	4.197	0.0158	6.077	0.0003341	0.1699
	120	1.501	0.0001888	0.0148	0.06637	0.869	0.01462	3.038
	130	1.461	0.0002069	16.76	0.06004	0.7417	0.01373	3.093
	140	1.45	0.0001821	10.53	0.1238	1.318	0.01485	2.638
NiCoP-2/NF	60	1.236	0.00074	3.389	0.01381	18.63	0.0001163	0.159
	70	1.227	0.0798	0.3938	0.01115	15.55	0.000124	0.1633
	80	1.227	0.0007577	0.3663	0.01017	11.72	0.001195	0.1555
	90	1.233	0.0009303	0.4437	0.00942	10.58	0.0001362	1.792
	100	1.238	0.0009571	0.3944	0.00921	7.188	0.001198	0.1744
	110	1.257	0.0000939	0.162	0.06932	2.29	0.007784	5.225
	120	1.252	0.0000936	0.1253	1.03	3.291	0.008612	4.236
NiCoP-3/NF	60	1.468	0.00133	0.3313	0.02364	17	0.0002515	0.0143
	70	1.455	0.01786	0.3479	0.02154	14.74	0.0003675	0.1483
	80	1.447	0.001797	0.3739	0.01998	11.49	0.0002763	1.768
	90	1.504	0.002	0.407	0.0183	9.552	0.0003439	0.0172
	100	1.442	0.00153	4.031	0.01712	6.938	0.0002953	0.1557
	110	1.446	0.001962	4.197	0.0158	6.077	0.0003341	0.1699
	120	1.501	0.0001888	0.0148	0.06637	0.869	0.01462	3.038
	130	1.461	0.0002069	16.76	0.06004	0.7417	0.01373	3.093
	140	1.45	0.0001821	10.53	0.1238	1.318	0.01485	2.638
NiP/NF	60	2.018	0.0006935	0.4838	0.00945	29.39	0.0001325	0.2264
	70	1.971	0.00068	5.082	0.00828	25.97	0.0001072	0.2354
	80	2.037	0.000142	0.0230	0.00835	24.51	0.001177	0.3027
	90	2.028	0.0001264	2.158	0.00873	22.75	0.00109	0.2947
	100	1.92	0.0006831	0.6051	0.00728	16.7	0.0001152	2.494
	110	2.008	0.0004933	0.4984	0.00758	16.39	0.0000096	0.1831
	120	1.907	0.006062	4.804	0.00712	12.06	0.0001191	0.2291
	130	1.952	0.0001184	0.1882	0.1699	10.39	0.006112	7.804
	140	1.964	0.001129	0.0615	0.2618	23.65	0.00574	6.091

References

1. Yu, L.; Mishra, I. K.; Xie, Y.; Zhou, H.; Sun, J.; Zhou, J.; Ni, Y.; Luo, D.; Yu, F.; Yu, Y.; Chen, S.; Ren, Z., Ternary $Ni_{2(1-x)}Mo_{2x}P$ nanowire arrays toward efficient and stable hydrogen evolution electrocatalysis under large-current-density. *Nano Energy* **2018,** *53*, 492-500.
2. Yu, C.; Xu, F.; Luo, L.; Abbo, H. S.; Titinchi, S. J. J.; Shen, P. K.; Tsiakaras, P.; Yin, S., Bimetallic Ni–Co phosphide nanosheets self-supported on nickel foam as high-performance electrocatalyst for hydrogen evolution reaction. *Electrochimica Acta* **2019,** *317*, 191-198.
3. Li, Q.; Chen, C.; Luo, W.; Yu, X.; Chang, Z.; Kong, F.; Zhu, L.; Huang, Y.; Tian, H.; Cui, X.; Shi, J., In situ active site refreshing of electro-catalytic materials for ultra-durable hydrogen evolution at elevated current density. *Advanced Energy Materials* **2024,** 1614-6832.
4. Zhai, P.; Zhang, Y.; Wu, Y.; Gao, J.; Zhang, B.; Cao, S.; Zhang, Y.; Li, Z.; Sun, L.; Hou, J., Engineering active sites on hierarchical transition bimetal oxides/sulfides heterostructure array enabling robust overall water splitting. *Nature Communications* **2020,** *11* (1)**,** 5462.
5. Liu, W.; Wang, X.; Wang, F.; Du, K.; Zhang, Z.; Guo, Y.; Yin, H.; Wang, D., A durable and pH-universal self-standing MoC–Mo_2C heterojunction electrode for efficient hydrogen evolution reaction. *Nature Communications* **2021,** *12* (1)**,** 6776.
6. Luo, Y.; Tang, L.; Khan, U.; Yu, Q.; Cheng, H.-M.; Zou, X.; Liu, B., Morphology and surface chemistry engineering toward pH-universal catalysts for hydrogen evolution at high current density. *Nature Communications* **2019,** *10* (1)**,** 269.
7. Jiang, S.; Zhang, R.; Liu, H.; Rao, Y.; Yu, Y.; Chen, S.; Yue, Q.; Zhang, Y.; Kang, Y., Promoting formation of oxygen vacancies in two-dimensional cobalt-doped ceria nanosheets for efficient hydrogen evolution. *Journal of the American Chemical Society* **2020,** *142* (14), 6461-6466.
8. Liu, R.; Gong, Z.; Liu, J.; Dong, J.; Liao, J.; Liu, H.; Huang, H.; Liu, J.; Yan, M.; Huang, K.; Gong, H.; Zhu, J.; Cui, C.; Ye, G.; Fei, H., Design of aligned porous carbon films with single-atom Co-N-C sites for high-current-density hydrogen generation. *Advanced Materials* **2021,** *33* (41), 2103533.
9. Senthil Raja, D.; Chuah, X. F.; Lu, S. Y., In situ grown bimetallic mof-based composite as highly efficient bifunctional electrocatalyst for overall water splitting with ultrastability at high current densities. *Advanced Energy Materials* **2018,** *8* (23), 1801065.
10. Liu, X.; Zheng, L.; Han, C.; Zong, H.; Yang, G.; Lin, S.; Kumar, A.; Jadhav, A. R.; Tran, N. Q.; Hwang, Y.; Lee, J.; Vasimalla, S.; Chen, Z.; Kim, S. G.; Lee, H., Identifying the activity origin of a cobalt single-atom catalyst for hydrogen evolution using supervised learning. *Advanced Functional Materials* **2021,** *31* (18), 2100547.
11. Liu, H.; Li, X.; Chen, L.; Zhu, X.; Dong, P.; Chee, M. O. L.; Ye, M.; Guo, Y.; Shen, J., Monolithic Ni-Mo-B bifunctional electrode for large current water splitting. *Advanced Functional Materials* **2021,** *32* (4), 2107308.
